# Integrating transcriptomics and metabolomics to characterise the response of *Astragalus membranaceus* Bge. var. *mongolicus* (Bge.) to progressive drought stress

**DOI:** 10.1186/s12864-016-2554-0

**Published:** 2016-03-05

**Authors:** Xin Jia, Chuangshu Sun, Yongchun Zuo, Guangyue Li, Guobin Li, Liangyu Ren, Guilin Chen

**Affiliations:** College of Life Science, The Good Agriculture Practice Engineering Technology Research Center of Chinese and Mongolian Medicine, Inner Mongolia University, Hohhot, 010021 China; The Key Laboratory of National Education Ministry for Mammalian Reproductive Biology and Biotechnology, Key Laboratory of Herbivore Reproductive Biotechnology and Breeding Ministry of Agriculture, Inner Mongolia University, Hohhot, 010070 China

**Keywords:** Drought, Metabolism, Gene expression, RNA-seq, *A. mongolicus*

## Abstract

**Background:**

*Astragalus membranaceus* Bge. var. *mongolicus* (Bge.) Hsiao (*A. mongolicus*) is an important traditional Chinese herb that is cultivated on a large scale in northwestern China. Understanding plant responses to drought has important effects on ecological environment recovery and local economic development. Here, we combined transcriptomics (Illumina Hiseq 2000) and metabolomics (^1^H-NMR) to investigate how the roots of two-year-old *A. mongolicus* responded to 14 days of progressive drought stress.

**Results:**

The dried soil reduced the relative water content (RWC) of the leaves and biomass, induced the differential expression of a large fraction of the transcriptome and significantly altered the metabolic processes. PCA analysis demonstrated that the sucrose, proline, and malate metabolites contributed greatly to the separation. Strikingly, proline was increased by almost 60-fold under severe stress compared to the control. Some backbone pathways, including glycolysis, tricarboxylic acid (TCA) cycle, glutamate-mediated proline biosynthesis, aspartate family metabolism and starch and sucrose metabolism, were significantly affected by drought. An integrated analysis of the interaction between key genes and the altered metabolites involved in these pathways was performed.

**Conclusions:**

Our findings demonstrated that the expression of drought-responsive genes showed a strong stress-dose dependency. Analysis of backbone pathways of the transcriptome and metabolome revealed specific genotypic responses to different levels of drought. The variation in molecular strategies to the drought may play an important role in how *A. mongolicus* and other legume crops adapt to drought stress.

**Electronic supplementary material:**

The online version of this article (doi:10.1186/s12864-016-2554-0) contains supplementary material, which is available to authorized users.

## Background

Plants colonised the land over 400 million years ago. Thereafter, they successfully evolved to cope with water deficits. For instance, some plants have an excellent tolerance to water scarcity during drought [1, 2]. The physiology, biochemistry, and molecular biology of plants are affected by some responses [3]. When faced with mild soil water stress, plants maintain their turgor pressure for a short time to ensure steady growth [4]. However, under mild to severe drought stress, plants show decreased photosynthesis and employ several measures to minimise water loss [5–7], including stomata closure and leaf rolling [8, 9], biomass desiccation [10], cell turgor maintenance, and osmotic adjustment [11]. Ultimately, the physiological responses to water stress are underpinned by reprogramming of the metabolism and gene expression [12–14]. Therefore, we need to elucidate these protective mechanisms to better understand how plants adapt to drought conditions.

Drought resistance is a complex process that is regulated by interactions among multiple genes [15]. Therefore, manipulation of a single gene or a subset of genes is not sufficient to study drought resistance. Instead, the complex responses of plants to drought stress need to be considered on a systems biology scale. Over the past decade, technological innovations have made it possible to overview the changes that occur at the transcriptomic level under environmental stress conditions. Many published studies have reported the use of microarrays and RNA sequencing techniques to identify changes in genes induced by drought treatments in a variety of plant species [16–21]. These results primarily identified genes that were up-regulated and/or down-regulated under drought stress. However, the plant response to drought is affected not only by the drought-responsive transcriptome but also by interactions among genes, metabolites and proteins. The relationship between the transcriptome and metabolome under water stress conditions has been characterised in different species [22–24].

The response of plants to soil water deficits may be governed by changes in small molecules to reach a new state. A number of previous reports have described metabolomics responses of plants to water and/or salt stress [25–30]. Several compounds synthesised by plants, such as sugars, ferulic acid, amino acids, and ABA have shown tolerance to drought stress [31, 32]. Osmotic adjustment via the accumulation of these solutes is one of the principal mechanisms by which plants adapt to water stress [33, 34]. With the reduction in osmotic potential, the plants can maintain cell turgor pressure and growth by changing the turgor-raising potential of the existing cellular water [35]. Some compatible solutes, such as mannitol, raffinose, polyols, trigonelline and proline, can reduce the osmotic potential [36, 37]. The trigonelline content increased when plants were subjected to stress, such as salt stress or drought stress [38, 39]. In many higher plants, proline accumulation was correlated with a water deficit [22, 40, 41]. These compounds also protect membranes and enzymes [37] during the drought process. Hence, we can better understand the mechanisms underlying plant resistance to drought stress by combining transcriptomics and metabolomics approaches.

The shortage of the water resource is a common phenomenon in Northwestern China and seriously hampers development and production. Therefore, we need to search for drought-adapted plants and study plant responses to drought stress to effectively tackle the problem. *Astragalus membranaceus* Bge. var. *mongolicus* (Bge.) Hsiao (*A. mongolicus*) is a traditional Chinese herb that has successfully evolved in arid and semi-arid environments. At present, it is primarily cultivated on a large scale by farmers in the northern and northwestern regions of China due to the shortage of wild resources. Understanding the herb resistance to drought stress has contributed to guiding planting and optimising the yield.

The purpose of this study is to present a comprehensive overview of the changes in the *A. mongolicus* transcriptome and metabolome under progressive drought stress and to identify important pathways through which this herb adapts to and tolerates drought stress. Moreover, we provide candidate genes that may provide useful information for translational approaches to investigate *A. mongolicus* or other legume crops in future studies.

## Results

### Physiological changes during drought acclimation

**Fig. 1:**
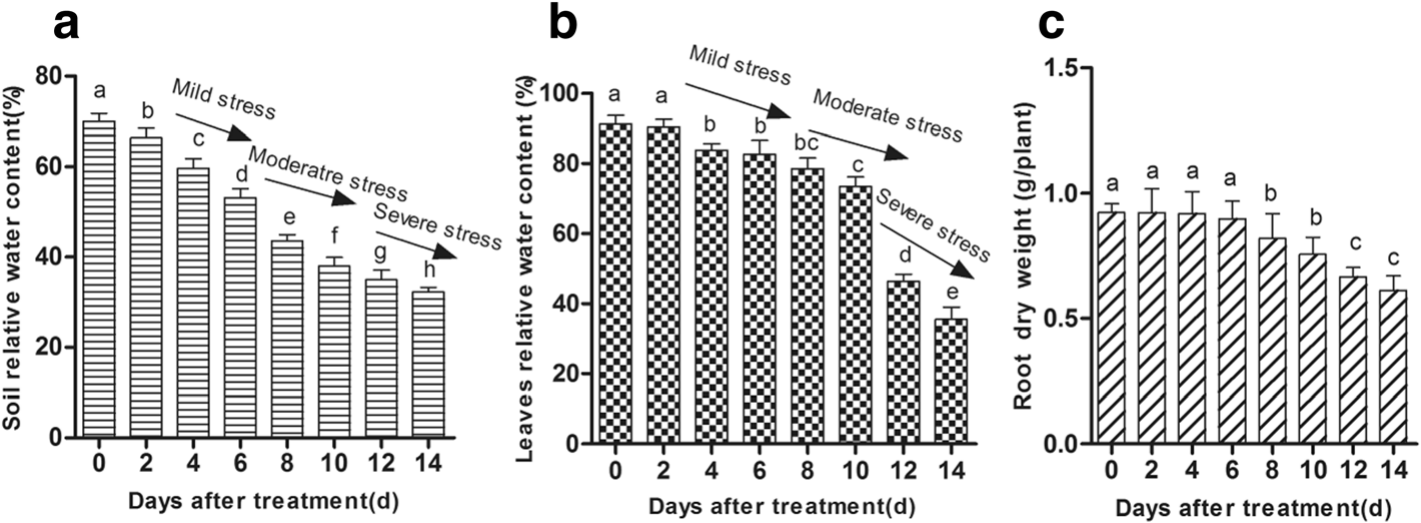
Physiological changes of *A. mongolicus* during progressive drought stress. **a** Effects of drought stress on soil water content (SWC). **b** Effects of drought stress on relative water content (RWC) of leaves. **c** Effects of drought stress on root dry weight (*n* = 8)

We grew the plants for 14 days while withholding water to make the drought stress treatments as realistic as possible. First, we tested the soil field capacity under drought stress. The soil relative water content (SWC) rapidly decreased from 70.0 % to 53.1 % on the 6^th^ day of the experiment. Then, the SWC gradually declined from 53.1 % to 32.3 % on the 14^th^ day (Fig. 1a). The relative water content (RWC) of leaves started to decline on the 4^th^ day and then it gradually declined to 73.5 % on the 10^th^ day. Following water withholding, the RWC of the leaves substantially decreased to 35.5 % on the 14^th^ day of the experiment (Fig. 1b).

We defined three phases of progressing drought stress according to the literature [41], the RWC, and phenology. During the mild stress stage (days 4–6), the RWC of the leaves declined by 8 %–10 % (Fig. 1b) without affecting the visible signs of plant growth relative to the control. During moderate stress (days 8–10), the soil water content decreased to 38.0 % (Fig. 1a). Although the RWC of the leaves remained higher than 50 % (Fig. 1b), the leaves were withered. The effects of severe stress (days 12–14) were quite intense. On the 14^th^ day, most plants exhibited yellowing and rolling of the leaves with a very low RWC (35.5 %). Furthermore, drought stress caused a significant reduction in the root dry weight (Fig. 1c). Taken together, these results indicated that drought stress had a significant impact on *A. mongolicus*. We performed transcriptomic and metabonomic analyses to elucidate the effects of drought stress on *A. mongolicus*. Based on the physiological changes under drought stress, we chose the following key time points for the study: 0d (A), 6d (B), 10d (C), and 14d (D).

### Transcriptomic analysis of *A. mongolicus* under progressive drought stress

**Fig. 2:**
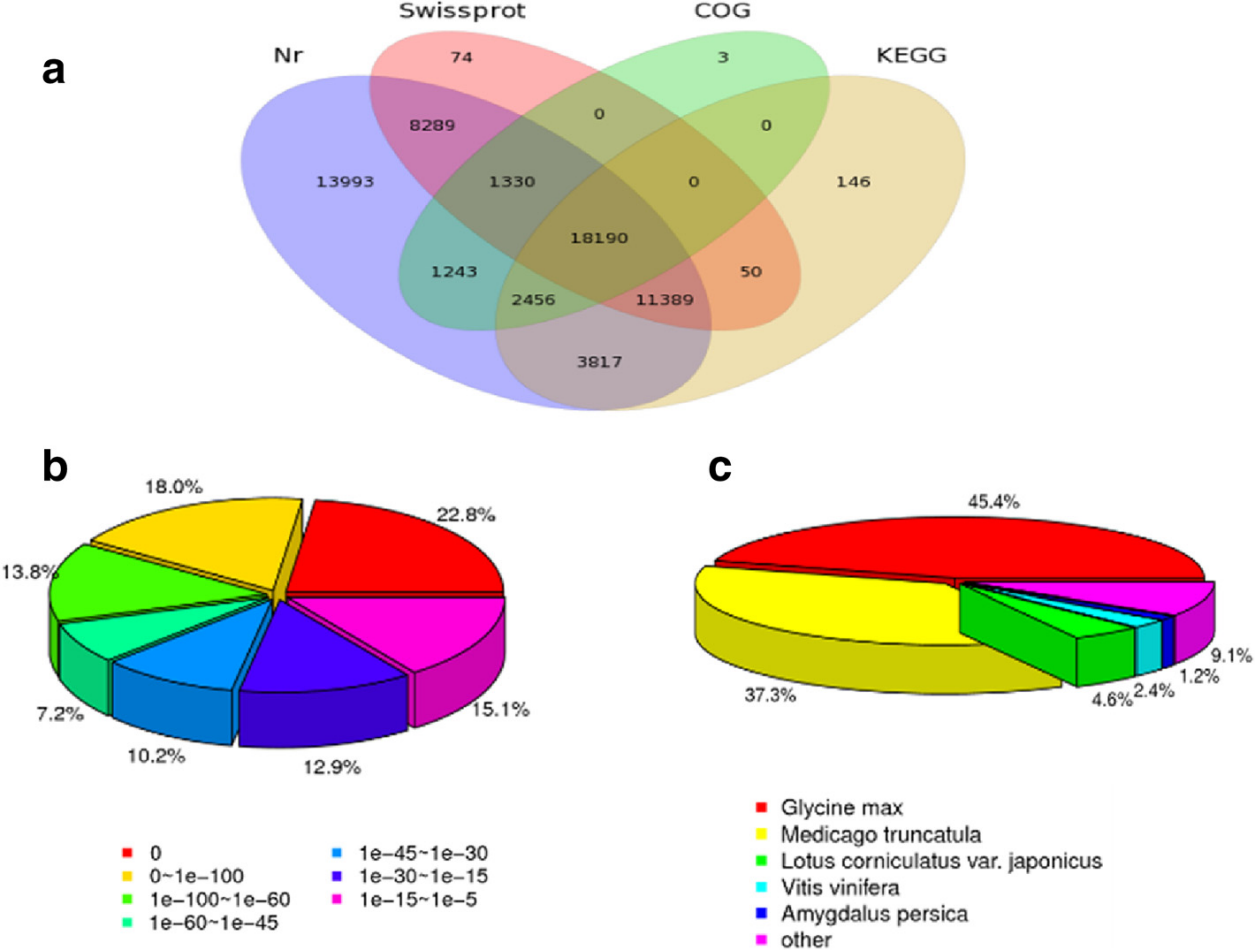
Distribution of the homology search of unigenes of against the database. **a** Venn diagram of a number of annotated unigenes by Blastx against protein databases. **b** *E*-value distribution of Blastx hits for each unigene against the NR database. **c** Species distribution of unigenes matching the top species using Blastx in the NR database

We used the Illumina Hiseq 2000 technology to generate a reference transcriptome of *A. mongolicus* under drought stress. We generated 18,885,163,680 bp and detected 88,662 unigenes. Table 1 displayed a total of 55,424,432, 56,050,320, 55,691,302, and 55,972,712 raw reads that were obtained from the control and water stress treatments. After removing low quality sequences, 52,178,802, 52,783,838, 52,231,504 and 52,641,008 clean reads were retained and used for assembly, respectively. The Q20 values (sequencing error rate < 1 %) were greater than 97.82 %, and the GC percentages were 42.13, 42.25, 42.29, and 42.08 %, respectively (Table 1). These results demonstrated that the sequencing data were of sufficient quantity and quality to ensure accurate sequence assembly and adequate transcriptome coverage. Figure 2a showed 60,707 unigenes annotated in the nr database according to the Basic Local Alignment Search Tool (BLASTx) (*E*-value < 1e^−5^) (NCBI, Bethesda, MD, USA) and 39,322 unigenes annotated according to the Swiss-Prot protein database (Swissprot, The European Bioinformatics Institute, Cambridge, UK). Additionally, 36,048 unigenes were annotated according to the Kyoto Encyclopaedia of Genes and Genomes (KEGG, Kanehisa Laboratories, Kyoto, Japan) and 23,222 unigenes were annotated using the Cluster of Orthologous Groups of Protein (COG) database (NCBI, Bethesda, MD, USA). A total of 13,993 unigenes were annotated only in the nr database, 74 unigenes were assigned only in Swissprot, and 3 unigenes and 146 unigenes were annotated only by COG and KEGG, respectively. Approximately 29 % (18,190/60,850) of the unigenes were assigned to a homolog in all four databases (Fig. 2a).

**Table 1:** Summary statistics of sequencing results

Samples	Total raw reads	Total clean reads	Total clean nucleotides(nt)	Q20 percentage	N percentage	GC percentage
A	55,424,432	52,178,802	4,696,092,180	97.91 %	0.01 %	42.13 %
B	56,050,320	52,783,838	4,750,545,420	97.99 %	0.01 %	42.25 %
C	55,691,302	52,231,504	4,700,835,360	97.82 %	0.01 %	42.29 %
D	55,972,712	52,641,008	4,737,690,720	97.96 %	0.01 %	42.08 %

A control, B water-stress for 6 days, C water-stress for 10 days, D water-stress for 14 days, respectively

The *E*-values and species distributions were analysed by evaluating the matched unigenes from the returned Blastx results of the nr protein database. Based on the *E*-value distribution of the top hits in the nr databases, 61.8 % of the aligned sequences showed strong homology (<1e-45), moreover, 58.29 % of the homologous sequences ranged from 1e-45 to 1e-5 (Fig. 2b). The species distribution of the top Blast hits for the *A. mongolicus* transcriptome showed that these unigenes had the greatest numbers of matches with the genes of *Glycine max* (45.4), followed by *Medicago truncatula* (37.3), *Lotus corniculatus var. japonicas* (4.6), *Vitis vinifera* (2.4) and *Amygdalus persica* (1.2 %). The other 9.1 % of the unigenes had hits with other species (Fig. 2c). This result indicates that the transcriptome of *A. mongolicus* is more closely related to *Glycine max* than to other plant genomes in the current public databases.

### Functional analysis of DE genes

**Fig. 3:**
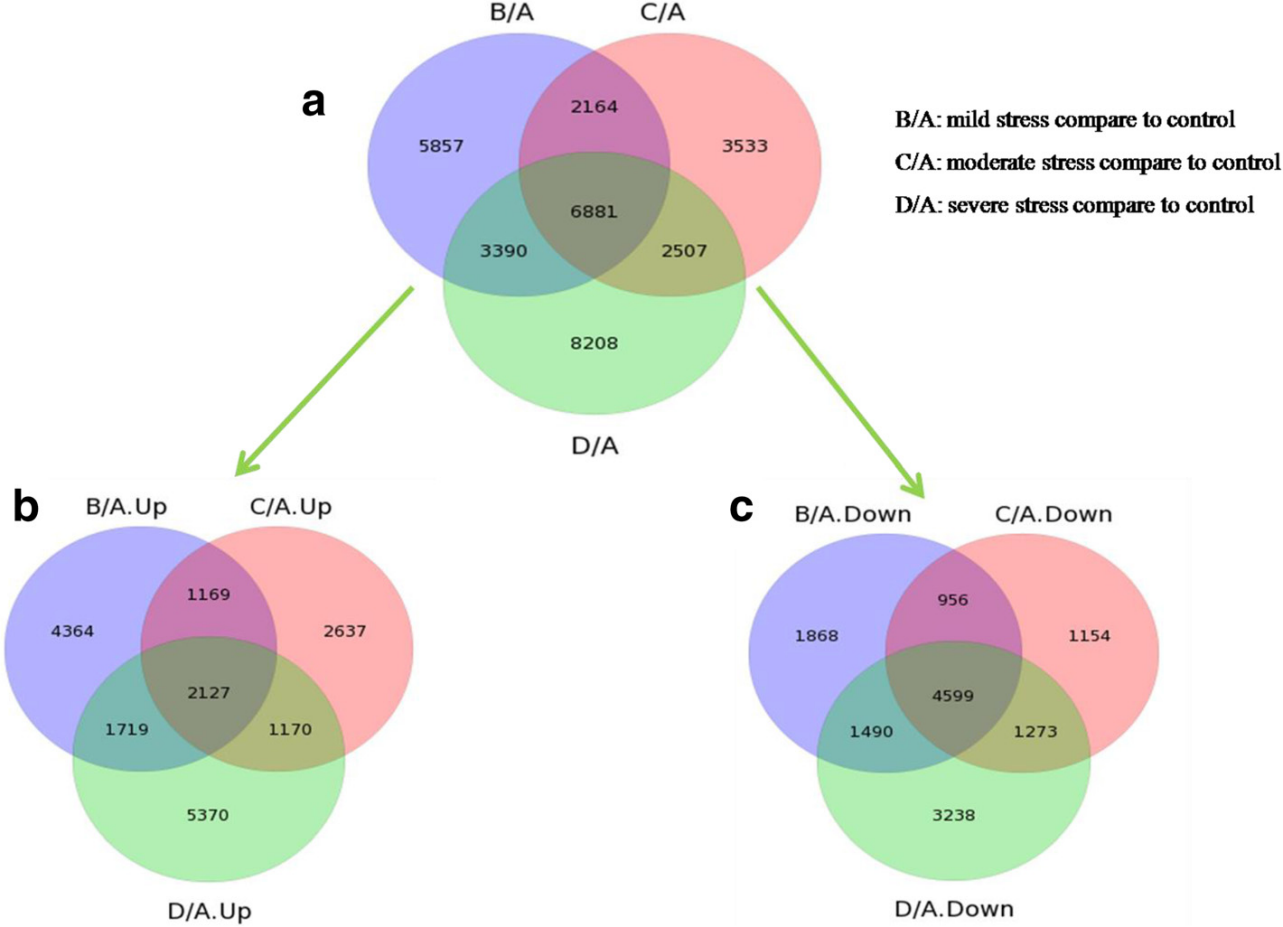
Transcriptomic analysis of *A. mongolicus* by high-throughput sequencing. **a** Venn diagram showing the number of differentially expressed (DE) genes, which were found at different treatment points. **b** Venn diagram showing the number of DE genes, which were only up-regulated at different treatment points. **c** Venn diagram showing the number of DE genes, which were only down-regulated at different treatment points (A: control, B: water stress for 6 days, C: water stress for 10 days, D: water stress for 14 days)

**Fig. 4:**
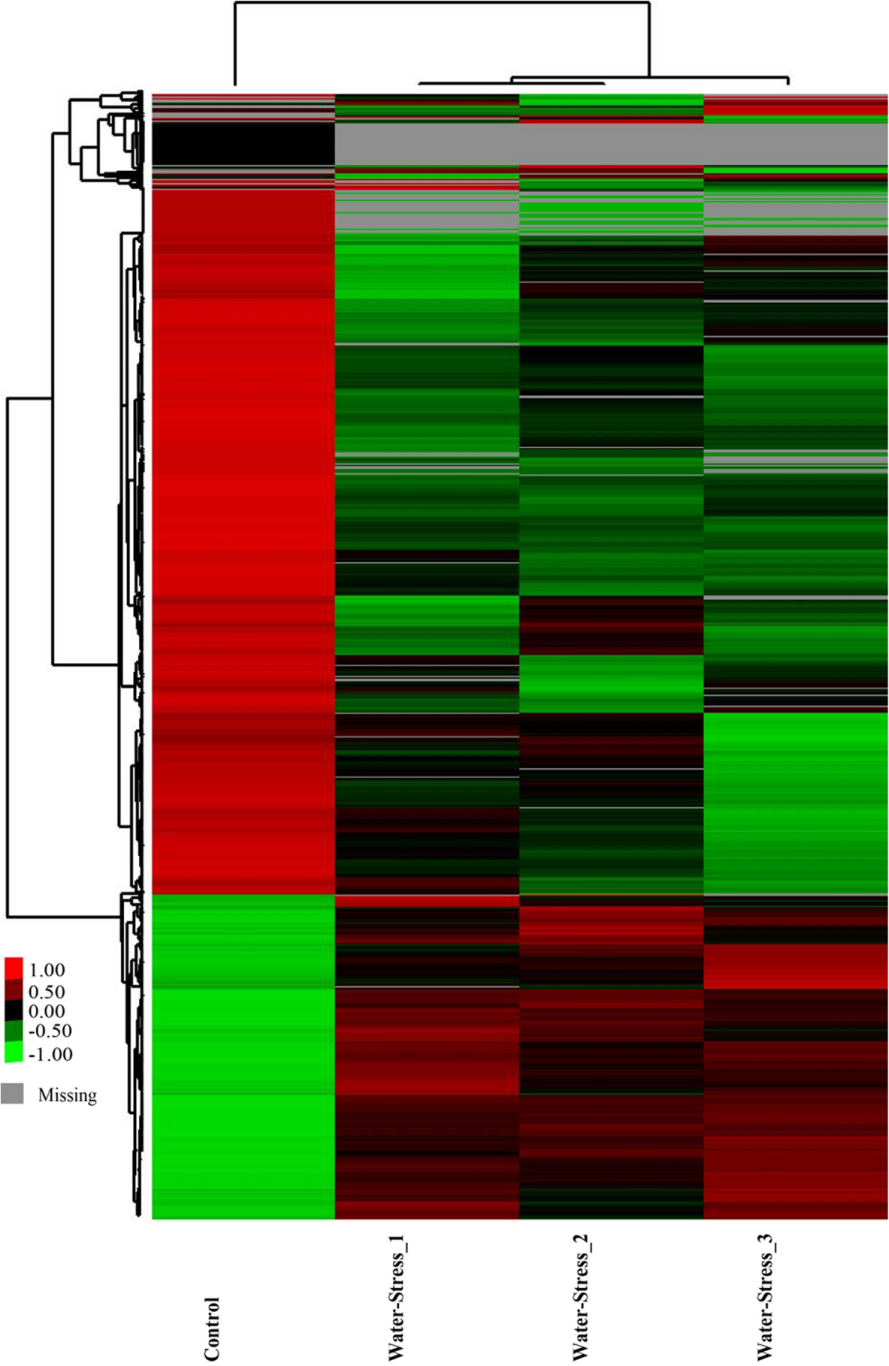
The hierarchical analyses of co-expression transcripts at different stages of drought stress. Green indicates the down-regulated and red indicates the up-regulated expression of genes (Control: 0 day, Water-stress-1: water stress for 6 days, Water-stress-2: water stress for 10 days, Water-stress-3: water stress for 14 days)

Functional analysis provides information and clarifies how *A. mongolicus* regulates its biological functions in response to drought stress at the molecular level. As shown in Fig. 3a, 18,292, 15,085, and 20,986 genes were differentially expressed on days 6, 10, and 14, respectively, compared to the control (0 day). However, only 6881 DE genes were co-expressed at all three levels of drought stress (2,127 DE genes were only significantly up-regulated and 4599 DE genes were only down-regulated) (Fig. 3b, c). We performed hierarchical clustering to obtain a complete transcriptional profile of the co-expressed transcripts under different stages of drought stress (Fig. 4). The clustered profiles indicated that drought stress significantly affected the transcriptional profiles of the co-expressed transcripts. The number of down-regulated genes was more than the number of up-regulated genes in these co-expressed transcripts compared to the control. Severe stress resulted in more down-regulated genes than the early stages of drought stress.

**Fig. 5:**
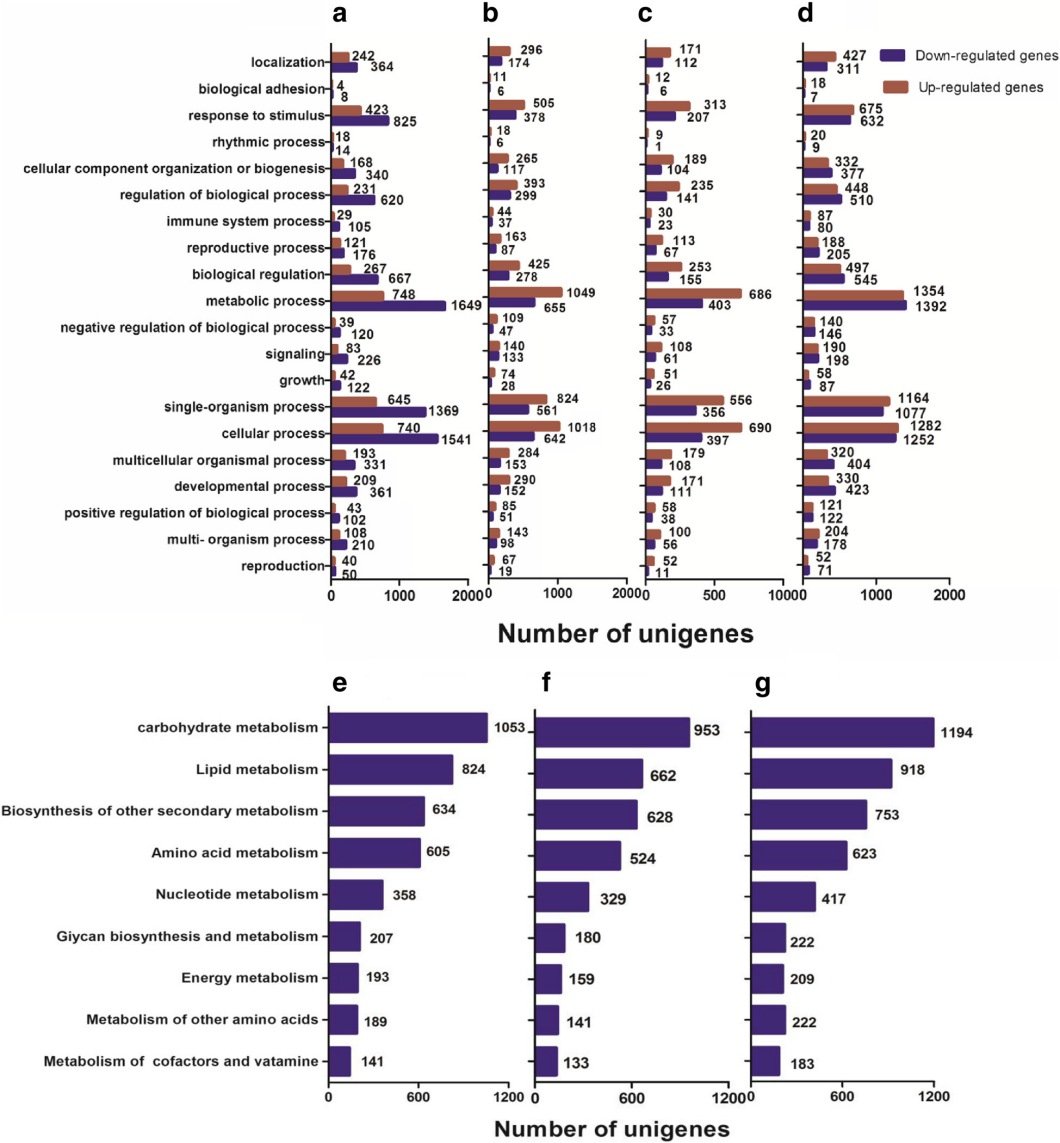
Plots of gene ontology (GO) terms from the biological process category of genes: **a** DE genes in total. **b** DE genes in mild stress (compare to the control). **c** DE genes in moderate stress (compare to the control). **d** DE genes in severe stress (compare to the control). **e** GO categories of a major metabolic process under mild stress (compare to the control). **f** GO categories of a major metabolic process under moderate stress (compare to control). **g** GO categories of a major metabolic process under severe stress (compare to control)

The DE genes were mapped to 20 gene ontology (GO) terms from the biological processing category (Fig. 5a, b, c, d). The most abundant DE genes were classified in the metabolism category, followed by cellular process and single-organism process. Interestingly, almost all of the related GO terms were enriched by the co-expressed down-regulated DE genes, such as genes related to growth, immunity and stimulus processes (Fig. 5a). The metabolism category contained more up-regulated DE genes than down-regulated DE genes under mild and moderate stress conditions (Fig. 5b, c). However, 1392 down-regulated genes were enriched under severe drought stress, resulting in a greater than 2-fold increase compared to mild stress (Fig. 5d). Additionally, the regulation of biological processes, signalling, and growth were more enriched in the down-regulated DE genes than the up-regulated genes (Fig. 5d). This finding indicates that severe stress can seriously affect the growth and regulation of biological processes in plants. Nine sub-categories were found under the metabolism category (Fig. 5e, f, g). In the metabolism category, the most genes were related to carbohydrate metabolism. A total of 1053 unigenes that mapped to carbohydrate metabolism were affected under mild stress conditions (Fig. 5e), 953 unigenes were affected by moderate stress (Fig. 5f), and 1,194 unigenes were affected by severe stress (Fig. 5 g). These changes were followed by genes related to lipid metabolism, secondary metabolism and amino acid metabolism, which were also affected by all stages of drought stress. The unigenes annotated in the metabolism category exercised their biological functions to catalyse metabolic processes or generate energy for primary and secondary metabolite production during progressive drought stress.

### Gene expression response to drought

Based on the expression ratio between mild stress relative to the control, we selected the 100 most highly expressed DE genes (Additional file 1: Table S1). Three of these highly elevated genes encoded *P5CS*, which was involved in the metabolism of the compatible solute proline. Two of the top 100 DE genes encoded a late embryogenesis abundant (LEA) protein. Interestingly, two genes were related to defensins, which influenced biotic stress [42]. Several genes encoded proteins of unknown function. Some of the top 100 highly expressed genes under severe stress (Additional file 2: Table S2) were related to ATP, three of these genes were auxin-regulated genes, whereas the functions of several genes were unknown.

We found that 35 DE genes were only up-regulated or only down-regulated during all three stages of drought stress. Among these genes, 25 genes were down-regulated DE genes (Additional file 3: Table S3). Furthermore, galactose metabolism was governed by two genes (the polygalacturonase gene and the fasciclin-like arabinogalactan gene). We also found one MADS-box transcription factor, two phosphoenolpyruvate carboxykinase (PEPC) genes, and one gene encoding cytochrome P450. However, 7 genes lacked functional annotation. In total, there were five enzymatic genes in this list, including a gene encoding a peroxidase enzyme, one probable gene encoding the carboxyl esterase 15-like enzyme, one gene encoding a methylmalonate-semialdehyde dehydrogenase and two genes encoding pectinesterase. Additionally, one gene was annotated as the pectate lyase enzyme. Other genes were also identified, including one auxin-induced gene, one formin-like gene, and two genes encoding laccase. Correspondingly, one gene might have encoded the toleration protein DRT100, which was probably associated with the repair of damaged DNA. Among these DE genes, the 10 up-regulated DE genes included one gene that probably encoded the U3 small nucleolar RNA, and one gene related to ATP. However, six genes had no functional annotation.

### Assignment of *A. mongolicus* metabolic changes during drought acclimation

To monitor the metabolic acclimation processes of *A. mongolicus* under drought stress, we performed nuclear magnetic resonance (NMR) in this study. Based on the ^1^H-NMR spectra of the methanol extracts from the *A. mongolicus* roots (Additional file 4: Figure S1), we identified sixteen amino acids and their derivatives, four carbohydrates, nine organic acids/amines, one nucleic acid component, three alcohols, and four other compounds (Additional file 5: Table S4). Sucrose was the highest out of the metabolites, and had a weight of > 30 mg per gram of freeze-dried plant at all time points. Other compounds, including asparagine, fructose, glucose and malate, had weights of >1 mg/g in the freeze-dried plant samples at all time points (Additional file 6: Table S5).

**Fig. 6:**
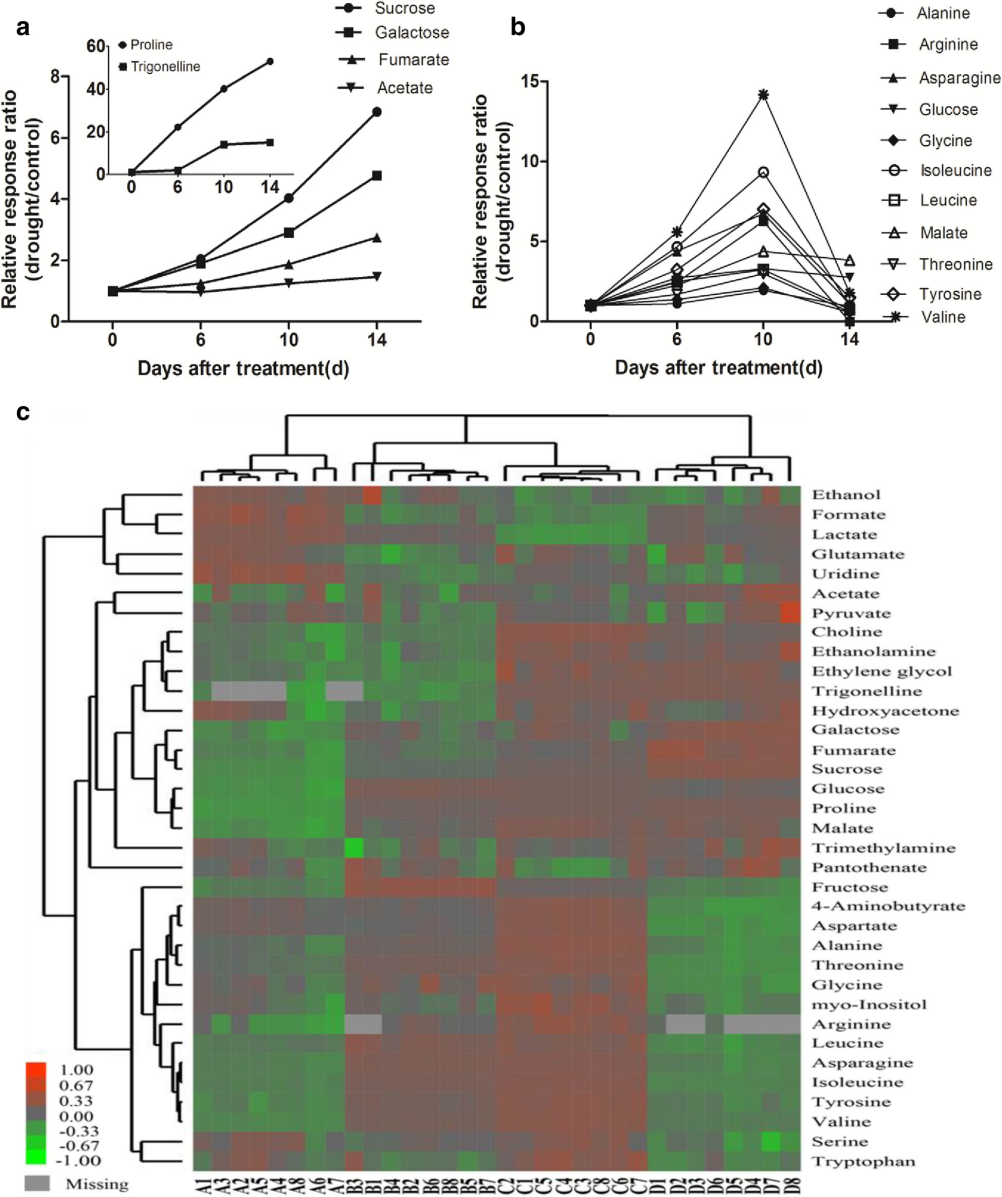
An overview of metabolic changes in *A. mongolicus*, which was exposed to drought stress. **a**, **b** Drought–induced metabolites concentration had relatively changed as compared to the control (0 day). **c** Differences in primary metabolite profiles during drought, heatmap color indicate the abundance of each metabolite in different stages of drought stress (A: control; B: water stress for 6 days; C: water stress for 10 days; D: water stress for 14 days)

During progressive drought stress, we found significantly changed metabolites based on the ratios of metabolite concentration changes to the control (Fig. 6a, b). Compared with the control, the most obviously changed metabolites included proline and trigonelline. The proline content was almost 60-fold higher during severe stress than in the control (Fig. 6a). Some other metabolites, such as galactose, ethanolamine, fumarate and acetate, also exhibited a strong stress-dose dependency. However, the levels of the other metabolites changed in a non-linear manner under water stress (Fig. 6b). For example, the concentration of most amino acids, such as valine, threonine, isoleucine, glycine, arginine, alanine, asparagines, leucine and tyrosine, the sugars fructose and glucose, and the organic acid malate, increased in response to the water deficit within 6 to 10 days reached an approximate plateau and then decreased to the control level on the 14^th^ day. Some metabolites, such as trimethylamine and pyruvate did not exhibt any significant changes in their concentrations (Additional file 6: Table S5). Based on the heat map analysis (Fig. 6c), we deduced that the abundant primary metabolites were significantly affected under drought stress. Most of the amino acids were significantly enriched under moderate stress, but dramatically decreased under severe stress. In contrast, some sugars, such as galactose, fumarate and sucrose, were increased under severe stress compared to the control.

**Fig. 7:**
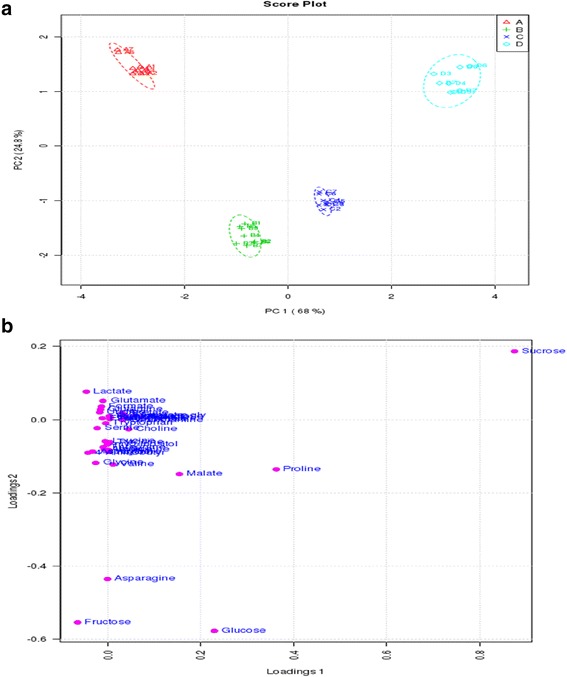
Metabolic analysis using a non-supervised principal component analysis (PCA). **a** Score plot of PCA. **b** Loading plot of PCA

We performed a multivariate analysis of the metabonomic responses. Principal component analysis (PCA) revealed the component data without prior metabolite identity information. The two component plot accounted for 93 % of the total variability. The first principal component accounted for 68 % of the variability, which was primarity reflected during mild and moderate drought stress, whereas the second principle component accounted for 24.8 % of the variability reflected control and severe drought stress (Fig. 7a). A stress-induced trend of dynamic changes in metabolites was clearly associated with the dosage of the drought stress. The PCA visualised sample classifications and implied good reproducibility of the ^1^H-NMR–based metabonomic method based on the trend of clustered samples. Moreover, from the loading plot (Fig. 7b), we inferred that sucrose, proline, malate, asparagine, fructose, and glucose greatly contributed to the separation of the different treatments.

### Integrative analysis of gene expression and metabolite levels

**Fig. 8:**
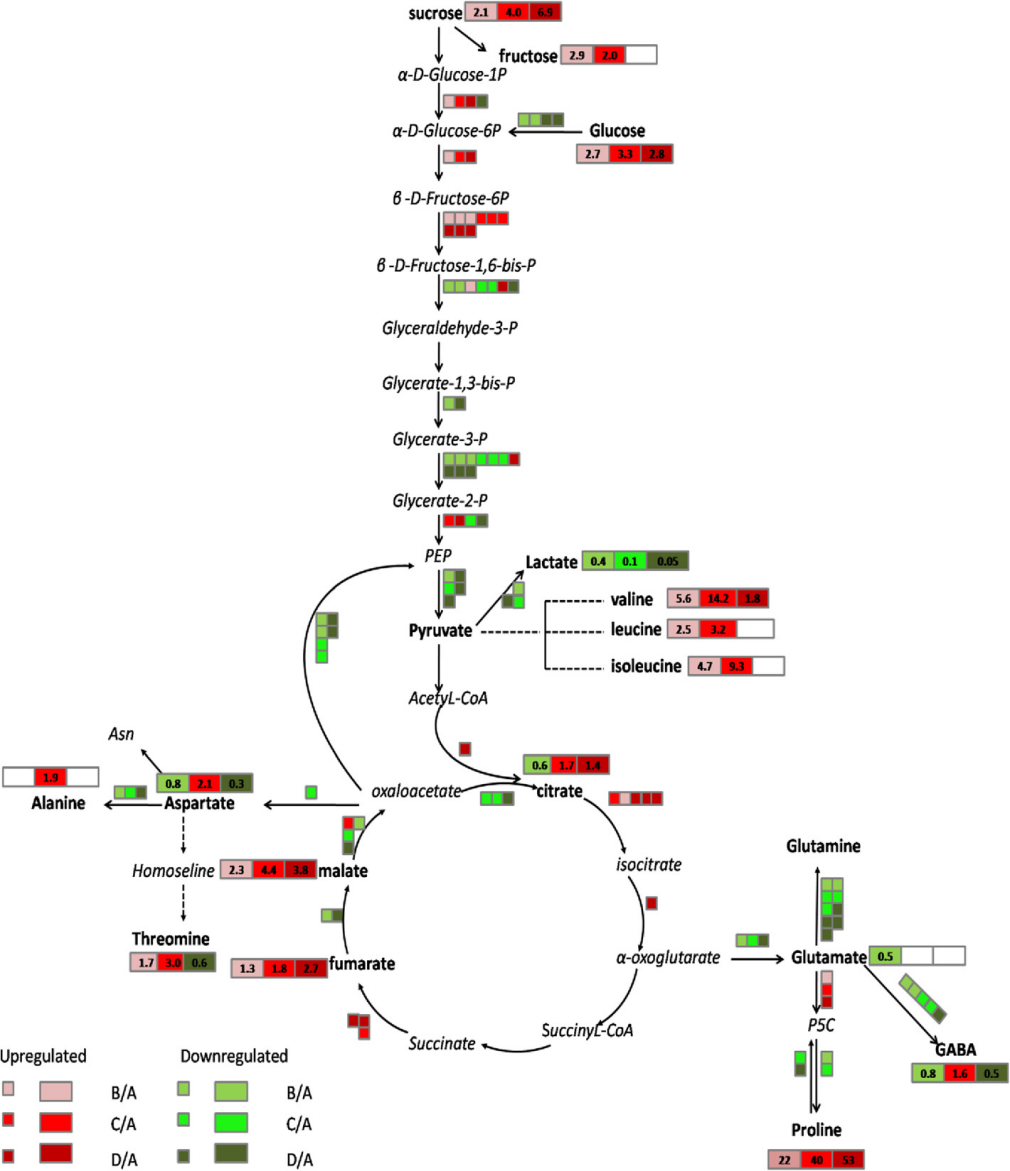
Pathway analysis related to *A. mongolicus* under progressive drought stress. The proposed metabolic pathways were based on literature and a web-based database of metabolic pathways. (A: control; B: water stress for 6 days; C: water stress for 10 days; D: water stress for 14 days). Metabolites, which were written in bold, were detected in this study. Metabolites in italic were not detected. Some metabolites have been omitted from this graph, because they were unaffected by drought stress. The small icons represent the genes, while the big icons represent the metabolites. Green indicates the down-regulated expression, while red indicates the up-regulated expression

We identified a relationship between gene expression and the abundance of metabolites. The list of metabolites, correlated genes, and primary metabolic pathways is provided in Table 2. We found that these pathways were significantly affected by drought stress. The major known pathways glycolysis, the tricarboxylic acid (TCA) cycle, glutamate-mediated proline biosynthesis and aspartate family metabolism were shown in the metabolism overview (Fig. 8).

**Table 2:** Annotation of differentially expressed genes associated with metabolites of *A. mongolicus* in response to drought

Pathway:Glycolysis		
Metabolites: Sucrose, Fructose, Glucose		
Gene ID	Gene annotation detail	*P*-value
*MTR8g104540*	Phosphoglucomutase	3.97E-10
*MTR1g102010*	Phosphoglucomutase	0
*MTR5g009000*	Hexokinase	3.29E-06
*MTR5g065880*	Glucose-6-phosphate isomerase	6.38E-19
*MTR6g090130*	6-phosphofructokinase	1.62E-26
*MTR091s0008*	6-phosphofructokinase	0
*MTR1g090300*	6-phosphofructokinase	3.13E-11
*MTR5g069050*	Fructose-bisphosphate aldolase	0
*MTR5g096670*	Fructose-bisphosphate aldolase	0.000103
*LOC100778356*	Phosphoglycerate kinase, chloroplastic-like	3.04E-07
*MTR7g074570*	2,3-bisphosphoglycerate-independent phosphoglycerate mutase	0
*MTR4g055260*	2,3-bisphosphoglycerate-dependent phosphoglycerate mutase	1.37E-09
*MTR145s0008*	2,3-bisphosphoglycerate-dependent phosphoglycerate mutase-like	1.47E-16
*MTR3g087540*	Pollen-specific protein C13	1.59E-14
*MTR5g096970*	Enolase	2.05E-06
*LOC101509496*	Heat shock factor binding protein	4.08E-07
*MTR7g100760*	Pyruvate kinase, cytosolic isozyme-like	6.14E-37
*MTR1g061630*	Pyruvate kinase	8.60E-14
*MTR5g014140*	Pyruvate kinase	6.07E-46
*MTR3g111120*	Phosphoenolpyruvate carboxykinase	2.78E-86
*MTR5g030620*	Phosphoenolpyruvate carboxykinase	2.08E-248
Pathway: Tricarboxylic acid (TCA)cycle		
Metabolites: Citrate, Fumarate, Malate		
*MTR5g091930*	Citrate synthase	8.17E-101
*MTR4g048190*	Aconitate hydratase	5.04E-19
*MTR1g075520*	Aconitate hydratase	3.58E-19
*MTR5g022940*	Aconitate hydratase	1.82E-45
*LOC101493754*	Isocitrate dehydrogenase [NAD] catalytic subunit 5	1.28E-09
*MTR5g020050*	Succinate dehydrogenase	0
*LOC101492705*	Succinate dehydrogenase	4.91E-51
*LOC101512712*	Fumarate hydratase 1, mitochondrial-like	1.24E-14
*MTR8g005980*	Malate dehydrogenase	3.78E-38
*MTR5g014710*	Malate dehydrogenase, glyoxysomal	1.96E-08
*MTR5g033920*	ATP-citrate synthase	4.24E-08
*MTR2g062920*	ATP-citrate synthase	6.30E-12
Pathway: Glutamate-mediated amino acids biosynthesis		
Metabolites: Glutamate, Glutamine, GABA, Proline		
*LOC101509615*	NADH-dependent glutamate synthase	3.91E-57
*MTR3g064740*	Glutamate decarboxylase	1.71E-79
*MTR3g064740*	Glutamate decarboxylase	1.70E-09
*MTR3g065250*	Glutamine synthetase	2.38E-05
*MTR5g077950*	Glutamine synthetase	2.69E-10
*LOC100780873*	Glutamine synthetase	6.70E-05
*MTR4g107940*	Delta-1-pyrroline-5-carboxylate dehydrogenase 1 protein	2.89E-115
*MTR7g020820*	Proline dehydrogenase	2.09E-13
Pathway: Aspartate family metabolisms		
Metabolites: Aspartate, Alanine, Threonine		
*MTR4g080140*	Aspartate aminotransferase	1.88E-14
*LOC101495687*	Peptide transporter PTR2-like	9.89E-18

Unigene sequences are aligned with @blastdb using blastx (evalue < 1e^−5^)

As shown in Fig. 8, genes encoding glycolytic enzymes, such as phosphoglucomutase, glucose-6-phosphate isomerase and phosphofructokinase, were upregulated during the three different phases of drought stress compared to the control. Regulation by phosphofructokinase is the most important step in glycolysis. During the metabolism of pyruvate and phosphoenolpyruvate (PEP), genes encoding pyruvate orthophosphatedikinase (PPDK) and phosphoenolpyruvate carboxylase are downregulated in the energy payoff phase of the glycolytic pathway. Genes encoding L-lactate dehydrogenase were decreased after drought stress relative to the control, which prevented the conversion of pyruvate into lactate. The regulation of glycolysis indicates that the changes in the abundance of adenosine triphosphate (ATP) may occur during different phases of drought stress. Each glucose molecule oxidised to pyruvate, which produce two ATP molecules. These ATP molecules provide energy for other physiological functions [43].

In the TCA cycle pathway, genes encoding aconitate hydratase were up-regulated during the three phases of drought stress. Genes related to succinate dehydrogenase were up-regulated under moderate and severe drought stress, whereas the genes encoding fumarate hydratase were down-regulated. These changes in gene expression may have increased the fumarate content by 2.7-fold compared to the control. Malate plays an important role in stress and usually responds to changes in stomatal conductance and osmotic potential [44, 45]. Using metabolomics analysis, we found that the malic acid content was increased under drought stress, whereas the expression of malate dehydrogenase (MDH) was down-regulated. The expression of genes involved in malate synthase was not consistent with the changes in the malic acid content (Fig. 8).

**Fig. 9:**
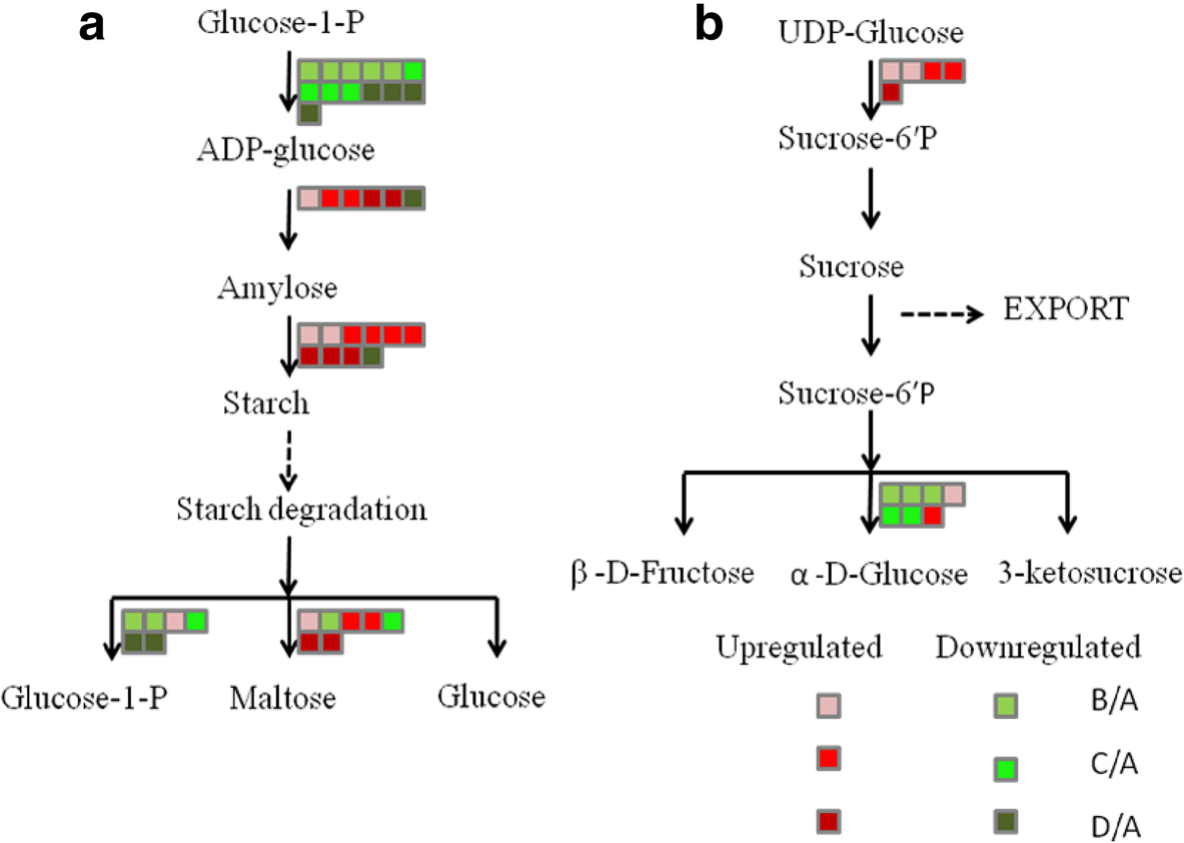
Changes in the expression of genes in starch and sucrose pathways of *A. mongolicus* under progressive drought stress. Altered expression of genes involved in starch metabolism (**a**) and sucrose metabolism (**b**). (A: control, B: water-stress for 6 days, C: water stress for 10 days, D: water stress for 14 days). Green indicates the down-regulated gene expression, while red indicates the up-regulated gene expression

The changes in the genes involved in glycolysis affected other key primary metabolic pathways, such as starch and sucrose metabolism. Most genes associated with starch synthesis were up-regulated under drought stress (Fig. 9), such as the genes encoding the starch branching enzyme. However, genes related to glucose-1-phosphate adenylyl transferase were down-regulated compared to the control. Sucrose metabolism was also affected. The expression of the sucrose-phosphate synthase *(SPS)* gene was up-regulated under drought stress and the invertase genes were down-regulated, which might be responsible for the progressive increase in sucrose in metabolomics results (Fig. 6a). These changes in key genes could alter the source–sink relationship in plants.

## Discussion

Our experiments were designed to study the response of *A. mongolicus* to drought stress. With a gradual water deficit, the plants showed significantly adaptive responses to stress. We determined the RWC of the soil and leaves during a progressive 14 day drought treatment. Based on the physiological and biochemical responses of *A. mongolicus* to drought stress, we imposed mild (6d), moderate (10d), and severe (14d) drought stress as references to reflect the stress intensity. Then, we gathered precise information from transcriptomics and metabolomics based on the drought intensity of the plants. We discussed the metabolic pathways and the correlated genes that were affected throughout the progressive drought stress.

The total number of drought-regulated genes was specifically correlated to the intensity of the imposed drought stress. However, we must note that drought tolerance-related genes may be activated earlier. For instance, most early response of genes may have participated in the stimulus response and growth process. We also found that most of the down-regulated genes participated in the signalling process. These genes were induced or repressed during the early stage of drought stress to enable the plant to adapt to the progressive water deficit. For instance, a gene encoding zeaxanthin epoxidase (ZEP) was detected under the mild stress condition and an up-stream enzyme of abscisic acid (ABA) was identified in the biosynthesis pathway. ABA is a major regulator of drought stress and can clearly trigger a wide range of avoidable mechanisms and physiological responses, including stomatal closure and osmoregulation [46, 47].

One of the consequences of stomatal closure due to progressive drought stress is the overproduction of reactive oxygen species (ROS), which cause oxidative stress. Transpirational cooling also leads to thermal stress. ROS can damage cells and the photosynthetic apparatus by oxidising amino acids and proteins [46, 48]. Our results indicated that the PSII (photosystem II) genes were down-regulated and several heat shock protein genes were induced after drought stress. These findings were consistent with previous studies on drought stress [49, 50] and demonstrated the plant responses to oxidative damage and thermal stress.

*Glycine max* and *A. mongolicus* are both legume crops but are not in the same genus. Our results showed that the *A. mongolicus* transcriptome had the largest number of matches with the genes of *Glycine max* (Fig. 2c). However, *Glycine max* and *A. mongolicus* showed different responses to water deficits. For instance, the growth and development of *Glycine max* are extremely sensitive to water deficits [51] with the exception of a few genotypes, whereas our results revealed that *A. mongolicus* exhibited good tolerance and adaption to drought stress. Chen et al. (2013) compared drought-tolerant and drought-sensitive *Glycine max* genotypes under dehydration and rehydration conditions and found that the genes encoding the auxin protein and zinc finger protein were up-regulated in the roots of the drought-tolerant genotype compared to the control. Zinc finger proteins play a central role in drought tolerance [52]. Our results also showed that genes in *A. mongolicus* related to these proteins were up-regulated under drought stress. Furthermore, cytochrome P450s can eliminate ROS, participate in photosynthesis, and serve as precursors to ABA synthesis [53]. They are more likely to determine drought stress tolerance [1]. In both drought-tolerant *Glycine max* and *A. mongolicus*, the expression of the genes encoding cytochrome P450s were suppressed after drought stress. These results indicated that *A. mongolicus* adapted to and tolerated to the drought.

Metabolite profiling can provide a view of the metabolic phenotypes. Thus, we can discover novel metabolic phenotypes not found by traditional targeted analyses and the identification of chemical signatures of specific phenotypes [54, 55]. A less biased NMR-based metabolomics approach was employed to determine a larger number of metabolites affected by drought stress. Our results identified the glycolysis, TCA cycle, glutamate-mediated proline biosynthesis, shikimate-mediated metabolism, and aspartate family metabolism pathways. The increase in the concentration of amino acids probably occurred due to the inhibition of protein biosynthesis or the increase in protein degradation induced by moderate drought stress. In the severe phase of drought stress, many amino acids decreased to the control level, probably due to secondary metabolism. Aromatic amino acids, such as tyrosine and phenylalanine are precursors for the biosynthesis of polyphenols and flavonoids. These compounds are natural secondary metabolites that act as endogenous antioxidants in the defence mechanisms of plants.

By comparing the metabolomics data with the transcriptomic data, we gathered more information about the metabolic pathways. Trigonelline and proline had striking regulatory profiles among the metabolites.

Trigonelline (TG) is an alkaloid that is found in the legume crop *Trigonella foenum graecum fabaceae* (fenugreek). TG also exists in other edible plants, such as peas, corn, and coffee. Because TG is an osmotic agent, its accumulation decreases the cellular osmotic potential, therefore TG plays an important role in protecting the stability of cell membranes when plants are subjected to stress [39]. Our results indicated that the TG content increased under mild stress and accumulated to a level that represented a 15-fold under severe stress compared to the control.

Proline has been widely considered to be a key drought-inducible metabolite because it plays an osmoprotective role in plants. As an osmotic agent, proline protects cell membranes by decreasing the cellular osmotic potential. Additionally, it has also been recognised as a regulator of the cellular redox status and a ROS scavenger [56–58]. In our study, we found that proline was one of the most significantly changed metabolites under different phases of drought stress. Proline increased from the 6^th^ day and reached its peak on the 14^th^ day of the experiment. Proline is synthesised from glutamate by the key enzyme 1-pyrroline-5-carboxylate synthetase (P5CS). Our study also revealed that *A. mongolicus* tightly regulated proline accumulation during progressive water stress by up-regulating the genes encoding P5CS. Two of the related genes (Unigene42840_All and CL3765.Contig3_All) exhibited very slight changes under drought stress compared to the control, whereas the expression of the third gene (CL8276.Contig2_All) was increased by four to five-fold under severe drought stress. Moreover, some of the identified genes encoded proline degrading enzymes, such as proline dehydrogenase (PDH). The down-regulation of the PDH gene occurred on the 6^th^ day of the experiment. Interestingly, proline was increased by 53-fold compared to the control under severe drought stress. However, the increase in gene expression was not as high as the proline content. This discrepancy suggests that proline synthesis may not occur first in the roots. Instead, proline may be first synthesised in the above-ground parts and then transported to the roots. Zhang [22] has that the proline content in the roots of the model legume forage *Medicago truncatula* was several fold higher than in the shoots, although the P5CS gene showed a higher expression level in the shoots under drought stress. Our results were also in agreement with the findings of Sharma’s [56] report.

In addition to these metabolites, soluble sugars often accumulate and perform functions such as signalling and osmotic adjustment [59]. In our study, we found that monosaccharide content (glucose and fructose) was increased and the genes involved in degradative metabolism were down-regulated under drought stress. Sugars can indirectly affect gene expression as an energy source. Additionally, sugars are direct precursors of metabolic reactions, such as osmotic regulators or signalling molecules [60]. The expression of invertase and its genes, which are involved in the conversion of amylose–starch and starch–maltose, were significantly affected by drought stress. These changes may significantly alter the communication of the source–sink.

In this study, metabolite profiling revealed an abundant accumulation of metabolites under drought stress. Moreover, we discovered pathways related to the tolerance and adaptation of *A. mongolicus* under drought stress conditions. We also elucidated the transcriptomic variation induced by different stages of drought stress. These findings provided an insight into the tolerance mechanisms of *A. mongolicus* and other legume crops subjected to drought stress.

## Conclusions

The findings of this study elucidate the mechanisms by which *A. mongolicus* responds to different stages of progressive drought stress. The data demonstrated the differential expression of several key genes, such as *P5CS, PDH, MDH*, zinc finger proteins genes, heat shock protein genes and cytochrome P450s genes, which are more likely to determine tolerance to drought stress, and identified the altered metabolites involved in the important glycolysis, TCA cycle, sucrose and starch metabolism, and glutamate-mediated proline biosynthesis pathways. These data may be used to develop methods to detect genes and metabolites of the host that respond to various stages of drought stress. Our findings help clarify the mechanism by which *A. mongolicus* and other legume crops tolerate and adapt to drought stress. Furthermore, by using various bioengineering approaches we can improve crop resistance to drought and optimise the yield. The findings of this study have a positive effect on solving the drought problem in arid and semi-arid areas.

## Methods

### Plant material and experimental design

The study was performed at Inner Mongolia University (39°40′ N, 110°52′ E, elevation 1040 m), Huhhot City, Inner Mongolia Province, Northwest China. *Astragalus membranaceus* Bge. var. *mongholicus* (Bge.) Hsiao seeds were collected from Wuchuan City, Inner Mongolia Province, China. The seeds were treated with concentrated sulfuric acid for 8 min and sterilised in 30 % bleach and 0.1 % Tween-20 for 10 min. Thereafter, the seeds were pre-germinated at 4 °C for 3d on a wet filter paper. On 30 March 2013, the germinated seeds were selected and sown in plastic pots (17 cm diameter × 25 cm height) filled with soil collected from the surface layer (0–30 cm) of a vegetable garden field located near the university. The soil pH was maintained at 7.5, and the soil contained 20.7 g/kg of organic matter, 114.7 mg/kg of hydrolysed nitrogen, 22.8 mg/kg of available phosphorus, and 207 mg/kg of available potassium. The plants were regularly watered and kept outside under natural conditions for one year. Each pot contained five seedlings. One month before the drought period, we chose healthy seedlings with an average height of 15.0 ± 0.4 cm. We moved these seedlings into a waterproof plastic shed. We weighed the pots and watered them every two days at 8:00 A.M.to maintain the field capacity conditions.

On 12 June 2014, the pots of seedlings were randomly divided into two groups: one group was watered well (control group) and the second group was not watered to simulate 14d of drought stress. During the experimental period, the average temperature was approximately 12 °C-18 °C at night and 22 °C-30 °C during the day. Light and relative humidity were maintained at constant levels. On days 0, 2, 4, 6, 8, 10, 12, and 14, six pots were randomly selected from each group and the seedlings were harvested at 8:00 A.M.. The harvested plants were kept in an ice box and immediately transported to the laboratory within five minutes. Three whole root samples were quickly cleaned frozen in liquid nitrogen and stored at −80 °C prior to RNA isolation. Furthermore, eight root samples were frozen in liquid nitrogen and stored at −20 °C prior to metabolite analysis. Other samples were used to measure the physiological indices. The RWC of the leaves was calculated using the formula:

$$ \mathrm{R}\mathrm{W}\mathrm{C}\ \left(\%\right) = \left[\left(\mathrm{F}\mathrm{W}\ \hbox{--}\ \mathrm{D}\mathrm{W}\right)/\left(\mathrm{S}\mathrm{W}\ \hbox{--} \mathrm{D}\mathrm{W}\right)\right] \times 100. $$

Here, FW represents the fresh weight of the leaf samples, SW represents the weight of leaves that were submerged in distilled water for 48 h, and DW represents the weight of leaves dried in an oven at 80 °C for 48 h. Eight root samples were washed with tap water and rinsed twice with distilled water. The samples were gently dried on a paper towel and then in a 60 °C oven for 48 h. Finally, we determined the dry weight of these leaves.

### RNA isolation and analysis

The *A. mongolicus* roots were ground into a fine powder for the total RNA isolation. From these powdered samples, we purified 200 ng of total RNA using oligo-dT beads. The poly (A)-containing mRNA was fragmented into small pieces using a one–cycle cDNA synthesis protocol. The purified and fragmented cDNA was combined with the End Repair Mix. Then, the end-repaired DNA was purified with Ampure XP Beads (AGENCOURT). Next, we added the A-Tailing Mix and mixed the samples well using a pipette. The adenylated 3′ ends of the DNA were combined with the RNA Index Adapter and Ligation Mix, and the reagents were mixed well by pipetting. Then, the end-repaired DNA was purified with Ampure XP Beads (AGENCOURT). We performed several rounds of PCR amplification using the PCR Primer Cocktail and PCR Master Mix to enrich the cDNA fragments. Then, the PCR products were purified using Ampure XP Beads (AGENCOURT).

The average length of the molecule was determined using the Agilent 2100 bioanalyser instrument (Agilent DNA 1000 Reagents). To quantify the library, we performed real-time quantitative PCR (QPCR) (TaqMan Probe). The qualified libraries were subjected to amplification using the cBot system, which generated the cluster on the flowcell (TruSeq PE Cluster Kit V3–cBot–HS, Illumina). The amplified flowcell was used for paired-end sequencing on a HiSeq 2000 System (TruSeq SBS KIT-HS V3, Illumina). Then, we determined that the average read length was 90 bp.

To analyse the correlations between gene functions, we performed a cluster analysis of the gene expression patterns using Cluster 3.0 and the JavaTreeview software. The Venn diagram and expression analysis of different transcripts were performed using R packages, GO term enrichment was calculated by comparing the number of annotations within the list of query transcripts to all of the annotated transcripts on the Poplar Affymetrix Genome Array. We characterised the important pathways associated with the drought transcriptome/metabolome using the Kyoto Encyclopaedia of Genes and Genomes (KEGG).

### Metabolomics profiling by ^1^H-NMR spectrometry

We prepared eight *A. mongolicus* root samples for the metabolomics investigation. The samples were separately ground into freeze-dried powder. Approximately 50 mg was extracted from each of these samples using 500 μL of water and 500 μL of methanol by vortexing for 1 min. Thereafter, the extracts were subjected to four seconds of ultrasound at three second intervals in one cycle for a total of eight cycles. Finally, we centrifuged the samples for 15 min at 13,000 rpm and 4 °C. The supernatants of all samples (700 μL) were transferred into a centrifuge tube and dried completely under nitrogen gas. Then, the residues were ground into a freeze-dried powder. Thereafter, 450 μL of water was added by vortexing for 30s, and 50 μL of a standard ACDSS was added to the sample by vortexing for 10s. This step was followed by centrifugation of the sample for 2 min at 13,000 rpm and 4 °C. The supernatants of all samples (480 μL) were transferred to standard NMR tubes for the ^1^H-NMR analysis.

All ^1^H-NMR spectra were collected at 25 °C on a 600 MHz Bruker spectrometer (AV III) equipped with an inverse cryoprobe. The ^1^H-NMR spectra were acquired using the first transient of the Bruker “noesygppr1d.comp” sequence. The spectra were collected using 32 transients at a recycle delay of 1 s. All free induction decay (FID) signals were input into the Chenomx NMR Suite Professional software (version 7.7, Chenomx, Edmonton, Canada). Then, the automatically adjusted phase was corrected to the baseline. The DSS-d6 groups were used as internal standard references while determining the chemical shift (set to 0 ppm) in all spectra; the convolution was reversed and adjusted based on the chemical shift index (CSI) and peak shape in the spectrum. According to the related information in the ^1^H-NMR spectra (chemical shift, peak shape, half band width, and coupling split classification), we used the DSS-d6 concentration and the peak area as standards. Finally, the Chenomx NMR suite software and an internal database (a standard Chenomx metabolite database) were used to perform qualitative and quantitative analyses of the NMR spectra.

### Statistical analysis

The multivariate statistical analysis and PCA were performed using the Chenomx NMR Suite 7.7 software. PCA was used to present the variance in the data matrix. Statistical significance was calculated using Fisher’s exact test in R. We adjusted the false recovery rate (FDR) in the transcriptome analysis by applying the Benjamini-Hochberg correction. Both t tests and ANOVA were performed using the SPSS software (version 19, IBM, Armonk, NY, USA). The tests were used to analyse the effects of water stress on the metabolite concentrations. Significant differences were declared at the level of *p* < 0.05.

### Ethics

All researches were carried out in accordance with institutional and local regulations. No special ethical consent or approval was required.

## Additional files

Additional file 1: Table S1. List of the top 100 highly expressed genes in the early phase of drought stress (day 6). (DOCX 19 kb) Additional file 2: Table S2. List of the top 100 highly expressed genes in the severe stage of drought stress (day 14). (DOCX 18 kb) Additional file 3: Table S3.. The genes that were only up-regulated or only down-regulated during the three stages of drought stress. (DOCX 17 kb) Additional file 4: Figure S1. A typical 600 MHz ^1^H NMR spectra of methanol extracts obtained from the *A. mongolicus* roots. (DOCX 256 kb) Additional file 5: Table S4. Raw metabolomics data under different stages of drought stress (*n* = 8). (CSV 13 kb) Additional file 6: Table S5. The metabolite content in the *A. mongolicus* roots under different stages of progressive drought stress. (DOCX 21 kb)

## Abbreviations

^1^H-NMR: ^1^H nuclear magnetic resonance
DE genes: Differentially expressed (DE) genes
DW: Dry weight
FDR: False discovery rate
FW: Fresh weight
GO: Gene ontology
P5CS: 1-pyrroline-5-carboxylatesynthetase
PCA: Principal component analysis
PEP: Phosphoenolpyruvate
PPDK: Pyruvate orthophosphatedikinase
PSII: Photosystem II
ROS: Reactive oxygen species
RWC: Relative water content
SW: Saturated weight
SWC: Soil water content
TCA: Citrate cycle
