# Supplementary material for: Integrating transcriptomics and metabolomics to characterise the response of Astragalus membranaceus Bge. var. mongolicus (Bge.) to progressive drought stress
Source: BMC Genomics. 2016 Mar 5;17:188. doi: 10.1186/s12864-016-2554-0 (PMC4779257; doi:10.1186/s12864-016-2554-0)
Supplement: Additional file 1: — Table S1. List of the top 100 highly expressed genes in the early phase of drought stress (day 6). (DOCX 19 kb) [file 12864_2016_2554_MOESM1_ESM.docx]

**Table S1** List of the top 100 highly expressed genes in the early drought stress phrase (day 6)

| GeneID | Fold change | Regulation | Annotation |
| --- | --- | --- | --- |
| Unigene15158_All | 229 | up | Defensin-like protein-like |
| Unigene22362_All | 200 | up | Unknown protein |
| CL10818.Contig2_All | 167 | up | Unknown protein |
| Unigene9663_All | 155 | up | 2-oxoisovalerate dehydrogenase subunit alpha |
| Unigene10452_All | 120 | up | Unknown protein |
| CL10830.Contig2_All | 113 | up | Delta-1-pyrroline-5-carboxylate synthetase 2 |
| Unigene6533_All | 110 | up | Transmembrane protein |
| CL10296.Contig1_All | 104 | up | Unknown protein |
| Unigene2088_All | 103 | up | Unknown protein |
| Unigene7071_All | 98 | up | Defensin |
| Unigene7659_All | 98 | up | Zeatin O-glucosyltransferase-like |
| Unigene26274_All | 97 | up | ABC transporter G family member 22-like |
| Unigene685_All | 96 | up | Unknown protein |
| Unigene13726_All | 92 | up | Integrase, catalytic region |
| CL8123.Contig2_All | 85 | up | Subtilisin inhibiton |
| CL10830.Contig1_All | 84 | up | Delta-1-pyrroline-5-carboxylate synthase |
| CL6382.Contig3_All | 84 | up | Unknown protein |
| CL1214.Contig3_All | 80 | up | NAC domain-containing protein 29-like |
| CL4627.Contig1_All | 79 | up | GRAM domain-containing protein 1B |
| Unigene992_All | 79 | up | Unknown protein |
| Unigene9548_All | 76 | up | Bidirectional sugar transporter SWEET14-like |
| Unigene3657_All | 75 | up | Unknown protein |
| CL11074.Contig2_All | 75 | up | Unknown protein |
| Unigene18882_All | 74 | up | Cyclin-A1-1-like |
| CL9830.Contig1_All | 74 | up | Glutathione S-transferase N-terminal domain containing protein |
| CL5772.Contig4_All | 73 | up | Expansin-like B1-like |
| CL3766.Contig1_All | 72 | up | Unknown protein |
| Unigene15572_All | 71 | up | Late embryogenesis abundant protein-2 |
| CL3186.Contig2_All | 71 | up | Unknown protein |
| Unigene16540_All | 69 | up | Unknown protein |
| Unigene1861_All | 69 | up | Unknown protein |
| Unigene24846_All | 63 | up | Unknown protein |
| CL3933.Contig1_All | 62 | up | Histone H2B-alpha-like |
| CL6869.Contig2_All | 58 | up | Dehydration-responsive element-binding protein 1F |
| CL3766.Contig4_All | 57 | up | Unknown protein |
| CL8981.Contig1_All | 56 | up | Unknown protein |
| Unigene5038_All | 56 | up | Zinc finger protein CONSTANS-LIKE 2-like |
| CL6382.Contig1_All | 55 | up | Unknown protein |
| Unigene722_All | 52 | up | Unknown protein |
| CL4578.Contig2_All | 52 | up | Unknown protein |
| Unigene7595_All | 50 | up | Unknown protein |
| Unigene27784_All | 49 | up | Unknown protein |
| Unigene27204_All | 47 | up | Delta-1-pyrroline-5-carboxylate synthase-like |
| Unigene5541_All | 47 | up | Unknown protein |
| CL5053.Contig1_All | 47 | up | Unknown protein |
| CL6382.Contig4_All | 46 | up | Unknown protein |
| Unigene3010_All | 44 | up | Unknown protein |
| Unigene28117_All | 44 | up | Unknown protein |
| Unigene16510_All | 43 | up | Unknown protein |
| CL11000.Contig9_All | 42 | up | Cellulose synthase A catalytic subunit 7 [UDP-forming]-like |
| CL993.Contig3_All | 41 | up | Dehydration-responsive element-binding protein 1F |
| Unigene25488_All | 40 | up | Unknown protein |
| Unigene21384_All | 40 | up | Unknown protein |
| Unigene5596_All | 40 | up | Unknown protein |
| CL10208.Contig2_All | 40 | up | Unknown protein |
| Unigene28080_All | 39 | up | Chromosome transmission fidelity protein 8 homolog |
| Unigene12625_All | 38 | up | Unknown protein |
| CL2149.Contig2_All | 37 | up | Photosystem I psaH protein |
| Unigene15466_All | 37 | up | Unknown protein |
| CL3186.Contig1_All | 37 | up | Unknown protein |
| CL6382.Contig2_All | 37 | up | Unknown protein |
| Unigene33276_All | 36 | up | Group 3 LEA protein |
| Unigene26640_All | 35 | up | Unknown protein |
| CL7530.Contig1_All | 35 | up | Solute carrier family 25 member |
| Unigene33289_All | 34 | up | Unknown protein |
| Unigene21202_All | 34 | up | Putative polyprotein, related |
| Unigene34675_All | 34 | up | Unknown protein |
| CL3927.Contig1_All | 32 | up | Unknown protein |
| Unigene11939_All | 32 | up | Dehydration-responsive element-binding protein 1F |
| Unigene24732_All | 32 | up | Unknown protein |
| Unigene15311_All | 32 | up | Ribonuclease H |
| Unigene25262_All | 32 | up | Unknown protein |
| Unigene23045_All | 32 | up | Gag/pol polyprotein |
| Unigene15132_All | 32 | up | Unknown protein |
| CL5786.Contig1_All | 32 | up | R2R3-MYB transcription factor |
| Unigene22391_All | 32 | up | Unknown protein |
| CL765.Contig2_All | 31 | up | Cyclic nucleotide-gated channel |
| CL5187.Contig7_All | 31 | up | Calmodulin-binding transcription activator 4-like |
| CL1787.Contig1_All | 31 | up | 17.9 kDa heat shock protein (hsp17.9) |
| Unigene34684_All | 31 | up | Unknown protein |
| Unigene28111_All | 31 | up | Unknown protein |
| Unigene35154_All | 31 | up | Unknown protein |
| CL501.Contig1_All | 30 | up | CRT/DRE binding factor 4 |
| CL11157.Contig4_All | 30 | up | Unknown protein |
| CL11313.Contig3_All | 30 | up | Unknown protein |
| Unigene10647_All | 30 | up | Unknown protein |
| Unigene15307_All | 30 | up | Unknown protein |
| Unigene20102_All | 30 | up | Unknown protein |
| CL4424.Contig7_All | 30 | up | Unknown proteinv |
| CL2192.Contig14_All | 29 | up | HVA22-like protein |
| Unigene12705_All | 29 | up | Unknown protein |
| Unigene8820_All | 29 | up | Vascular protein |
| CL9343.Contig1_All | 29 | up | Unknown protein |
| Unigene5893_All | 29 | up | Unknown protein |
| Unigene12618_All | 29 | up | Unknown protein |
| CL9288.Contig3_All | 29 | up | rubisco activase |
| CL1787.Contig3_All | 28 | up | 17.9 kDa heat shock protein (hsp17.9) |
| CL4430.Contig6_All | 28 | up | Rubisco activase |
| Unigene27805_All | 28 | up | Sodium/hydrogen exchanger 2-like |
| Unigene10441_All | 28 | up | Unknown protein |
