# Supplementary material for: Integrating transcriptomics and metabolomics to characterise the response of Astragalus membranaceus Bge. var. mongolicus (Bge.) to progressive drought stress
Source: BMC Genomics. 2016 Mar 5;17:188. doi: 10.1186/s12864-016-2554-0 (PMC4779257; doi:10.1186/s12864-016-2554-0)
Supplement: Additional file 2: — Table S2. List of the top 100 highly expressed genes in the severe stage of drought stress (day 14). (DOCX 18 kb) [file 12864_2016_2554_MOESM2_ESM.docx]

**Table S2** List of the top 100 highly expressed genes in the severe stage of drought stress (day 14)

| GeneID | Fold change | Regulation | Annotation |
| --- | --- | --- | --- |
| Unigene42040_All | 556 | up | Unknown protein |
| Unigene42389_All | 223 | up | Integrin-linked protein kinase |
| CL3402.Contig1_All | 200 | up | Membrane protein, putative |
| CL4035.Contig4_All | 195 | up | Unknown protein |
| CL180.Contig9_All | 180 | up | Probable protein phosphatase 2C 28-like |
| CL7546.Contig1_All | 160 | up | Unknown protein |
| CL3666.Contig6_All | 148 | up | Unknown protein |
| CL3071.Contig1_All | 148 | up | Unknown protein |
| Unigene42407_All | 129 | up | Hypothetical |
| Unigene42972_All | 125 | up | Unknown protein |
| Unigene42938_All | 121 | up | Unknown protein |
| Unigene42546_All | 119 | up | Unknown protein |
| CL6093.Contig2_All | 113 | up | ADP,ATP carrier protein, mitochondrial-like |
| Unigene40720_All | 107 | up | Polyprotein |
| Unigene41391_All | 102 | up | Unknown protein |
| Unigene41465_All | 100 | up | Unknown protein |
| CL7510.Contig1_All | 99 | up | Unknown protein |
| Unigene43033_All | 95 | up | Unknown protein |
| Unigene42377_All | 94 | up | Unknown protein |
| CL5999.Contig2_All | 92 | up | F-box protein |
| CL2545.Contig2_All | 91 | up | Unknown protein |
| Unigene42308_All | 89 | up | Unknown protein |
| Unigene43030_All | 86 | up | Unknown protein |
| CL303.Contig2_All | 85 | up | Unknown protein |
| CL2634.Contig3_All | 85 | up | Unknown protein |
| Unigene42027_All | 81 | up | Unknown protein |
| Unigene40675_All | 76 | up | Unknown protein |
| CL10307.Contig2_All | 75 | up | Unknown protein |
| CL589.Contig1_All | 75 | up | Unknown protein |
| Unigene20068_All | 74 | up | ADP,ATP carrier protein |
| Unigene42334_All | 73 | up | Unknown protein |
| CL3534.Contig2_All | 72 | up | Polyprotein |
| Unigene42939_All | 70 | up | Unknown protein |
| CL9447.Contig3_All | 69 | up | Unknown protein |
| CL4795.Contig2_All | 65 | up | Unknown protein |
| Unigene10156_All | 64 | up | Kunitz-type trypsin inhibitor-like 1 protein |
| Unigene41519_All | 63 | up | Unknown protein |
| Unigene42564_All | 63 | up | U-box domain-containing protein 35-like |
| CL6600.Contig3_All | 62 | up | DEAD-box ATP-dependent RNA helicase |
| CL10416.Contig2_All | 62 | up | Unknown protein |
| Unigene42313_All | 61 | up | Auxin efflux carrier protein |
| Unigene42366_All | 59 | up | Unknown protein |
| Unigene41158_All | 59 | up | NF-X1-type zinc finger protein NFXL1 |
| Unigene42940_All | 59 | up | Unknown protein |
| Unigene43113_All | 58 | up | Cysteine proteinase |
| Unigene42575_All | 57 | up | Unknown protein |
| Unigene42944_All | 57 | up | Unknown protein |
| CL10788.Contig2_All | 56 | up | Unknown protein |
| Unigene42978_All | 55 | up | Unknown protein |
| CL9672.Contig2_All | 52 | up | Unknown protein |
| Unigene17006_All | 52 | up | Unknown protein |
| CL221.Contig2_All | 52 | up | Hexose transporter |
| Unigene42299_All | 51 | up | RNA-directed DNA polymerase (Reverse transcriptase) |
| Unigene42421_All | 51 | up | Unknown protein |
| Unigene42453_All | 51 | up | Unknown protein |
| Unigene41455_All | 51 | up | LON peptidase N-terminal domain and RING finger protein 1-like |
| Unigene43094_All | 50 | up | Unknown protein |
| Unigene41739_All | 50 | up | Unknown protein |
| Unigene42884_All | 50 | up | Unknown protein |
| CL11228.Contig1_All | 49 | up | 6-hydroxynicotinate 3-monooxygenase-like |
| Unigene41207_All | 49 | up | Phosphatidylinositol transfer protein |
| CL10823.Contig1_All | 49 | up | Unknown protein |
| CL1833.Contig1_All | 48 | up | Unknown protein |
| Unigene42968_All | 47 | up | Unknown protein |
| Unigene13582_All | 46 | up | Unknown protein |
| CL4368.Contig4_All | 46 | up | Unknown protein |
| CL9084.Contig2_All | 45 | up | Unknown protein |
| CL1792.Contig2_All | 45 | up | Unknown protein |
| CL6639.Contig6_All | 45 | up | Hydroxyproline-rich glycoprotein family protein |
| CL4034.Contig2_All | 44 | up | Unknown protein |
| Unigene42434_All | 44 | up | Unknown protein |
| Unigene40709_All | 44 | up | Polygalacturonase inhibitor protein |
| CL4488.Contig2_All | 44 | up | DNA-directed RNA polymerase III subunit RPC2-like |
| Unigene43022_All | 43 | up | Ty3/gypsy retrotransposon protein |
| CL2959.Contig1_All | 43 | up | Unknown protein |
| Unigene42612_All | 42 | up | Embryonic abundant protein-like protein |
| Unigene14315_All | 42 | up | Unknown protein |
| CL3053.Contig4_All | 41 | up | Unknown protein |
| Unigene42533_All | 41 | up | Unknown protein |
| CL3056.Contig4_All | 40 | up | Unknown protein |
| CL11096.Contig1_All | 40 | up | Unknown protein |
| CL3355.Contig8_All | 39 | up | Isoprenylcysteine alpha-carbonyl methylesterase ICME-like |
| CL5491.Contig1_All | 39 | up | Unknown protein |
| Unigene43067_All | 39 | up | Probable protein phosphatase 2C 33-like |
| Unigene43123_All | 39 | up | Unknown protein |
| Unigene42999_All | 39 | up | Unknown protein |
| CL11120.Contig1_All | 39 | up | Unknown protein |
| Unigene19512_All | 39 | up | UDP-glycosyltransferase 85A2-like |
| CL4021.Contig4_All | 38 | up | LYR motif-containing protein 4B |
| CL2331.Contig3_All | 38 | up | SET domain-containing protein |
| CL9496.Contig1_All | 38 | up | Unknown protein |
| Unigene42301_All | 38 | up | Beta-galactosidase 3-like isoform 1 |
| Unigene43233_All | 38 | up | Integrin-linked protein kinase 57 |
| Unigene42315_All | 37 | up | Auxin efflux carrier protein |
| Unigene20950_All | 37 | up | Unknown protein |
| CL2394.Contig4_All | 37 | up | Unknown protein |
| CL9078.Contig1_All | 37 | up | Unknown protein |
| CL7806.Contig3_All | 36 | up | Auxin-induced protein 5NG4 |
| Unigene43295_All | 36 | up | Unknown protein |
| Unigene43222_All | 36 | up | Late embryogenesis abundant domain-containing protein |
