# Supplementary material for: Integrating transcriptomics and metabolomics to characterise the response of Astragalus membranaceus Bge. var. mongolicus (Bge.) to progressive drought stress
Source: BMC Genomics. 2016 Mar 5;17:188. doi: 10.1186/s12864-016-2554-0 (PMC4779257; doi:10.1186/s12864-016-2554-0)
Supplement: Additional file 3: — Table S3.. The genes that were only up-regulated or only down-regulated during the three stages of drought stress. (DOCX 17 kb) [file 12864_2016_2554_MOESM3_ESM.docx]

**Table S3** The genes that were only up-regulated or only down-regulated during the three stages of drought stress

| Expression | Rank | Probe sets | Gene annotation and locus | P-value |
| --- | --- | --- | --- | --- |
| Down-regulated | 1 | CL4162.Contig1_All | gi\|297746137\|emb\|CBI16193.3\| /unnamed protein product | 3.61E-45 |
|  | 2 | CL8463.Contig2_All | gi\|358249196\|ref\|NP_001239753.1\| uncharacterized protein | 1.64E-31 |
|  | 3 | Unigene23751_All | gi\|357491147\|ref\|XP_003615861.1\|Laccase-like multicopper oxidase | 5.22E-14 |
|  | 4 | CL495.Contig2_All | gi\|358348969\|ref\|XP_003638513.1\| Pectate lyase | 0 |
|  | 5 | Unigene28590_All | gi\|357461551\|ref\|XP_003601057.1\|/Auxin-induced protein 5NG4 | 0 |
|  | 6 | Unigene15013_All | gi\|357514859\|ref\|XP_003627718.1\|DNA-damage-repair/toleration protein DRT100 | 0 |
|  | 7 | Unigene15071_All | gi\|356513351\|ref\|XP_003525377.1\|probable polygalacturonase-like | 7.65E-294 |
|  | 8 | CL692.Contig3_All | gi\|357507917\|ref\|XP_003624247.1\|Formin-like protein | 5.52E-15 |
|  | 9 | CL9785.Contig1_All | gi\|356519367\|ref\|XP_003528344.1\|RNA-dependent RNA polymerase 1-like | 1.17E-20 |
|  | 10 | CL10489.Contig1_All | gi\|356547990\|ref\|XP_003542387.1\| pectinesterase/pectinesterase inhibitor PPE8B-like | 6.31E-33 |
|  | 11 | CL11092.Contig2_All | gi\|356516287\|ref\|XP_003526827.1\|uncharacterized protein | 3.13E-53 |
|  | 12 | Unigene24783_All | gi\|356507971\|ref\|XP_003522736.1\| phosphoenolpyruvate carboxykinase [ATP]-like | 1.41E-28 |
|  | 13 | Unigene9218_All | gi\|357476329\|ref\|XP_003608450.1\| hypothetical protein MTR_4g094240 | 0 |
|  | 14 | Unigene18484_All | gi\|357466799\|ref\|XP_003603684.1\| Phosphoenolpyruvate carboxykinase | 6.74E-31 |
|  | 15 | Unigene4673_All | gi\|356547990\|ref\|XP_003542387.1\| pectinesterase/pectinesterase inhibitor PPE8B-like | 3.88E-28 |
|  | 16 | Unigene21475_All | gi\|356555437\|ref\|XP_003546038.1\| uncharacterized protein | 9.94E-20 |
|  | 17 | Unigene5051_All | gi\|388505302\|gb\|AFK40717.1\| /unknown | 1.41E-27 |
|  | 18 | CL3113.Contig4_All | gi\|357446015\|ref\|XP_003593285.1\| Methylmalonate-semialdehyde dehydrogenase | 2.44E-42 |
|  | 19 | Unigene9588_All | gi\|356544564\|ref\|XP_003540719.1\| laccase-2-like | 1.88E-33 |
|  | 20 | Unigene3697_All | gi\|357453863\|ref\|XP_003597212.1\| Fasciclin-like arabinogalactan protein | 7.14E-60 |
|  | 21 | CL4774.Contig1_All | gi\|357438829\|ref\|XP_003589691.1\| Cytochrome P450 | 1.25E-28 |
|  | 22 | Unigene26904_All | gi\|356505457\|ref\|XP_003521507.1\| probable carboxylesterase 15-like | 4.57E-13 |
|  | 23 | Unigene27041_All | gi\|357453491\|ref\|XP_003597023.1\| Peroxidase | 7.39E-24 |
|  | 24 | Unigene8059_All | No annotation | 2.82E-17 |
|  | 25 | Unigene28164_All | gi\|356530917\|ref\|XP_003534025.1\| MADS-box transcription factor 6-like | 2.04E-167 |
| Up-regulated | 1 | CL10075.Contig4_All | gi\|356569796\|ref\|XP_003553082.1\| uncharacterized protein | 7.69E-101 |
|  | 2 | CL10208.Contig1_All | No annotation | 1.11E-27 |
|  | 3 | Unigene11933_All | No annotation | 0.000164 |
|  | 4 | CL4383.Contig1_All | gi\|357513003\|ref\|XP_003626790.1\|1aminocyclopropane -1-carboxylate oxidase-like protein | 3.33E-08 |
|  | 5 | CL5347.Contig2_All | gi\|82394646\|gb\|ABB72412.1\|seed maturation protein | 3.54E-39 |
|  | 6 | CL5428.Contig1_All | gi\|357511207\|ref\|XP_003625892.1\|/ hypothetical protein MTR_7g108440 | 5.19E-05 |
|  | 7 | CL5919.Contig2_All | No annotation | 2.38E-07 |
|  | 8 | CL8898.Contig4_All | gi\|356516065\|ref\|XP_003526717.1\| ATP-dependent Clp protease ATP-binding subunit ClpX-like | 1.33E-05 |
|  | 9 | Unigene26082_All | gi\|359486916\|ref\|XP_002269978.2\| probable U3 small nucleolar RNA-associated protein 7 | 7.58E-36 |
|  | 10 | Unigene38440_All | No annotation | 9.19E-34 |

Unigene sequences are aligned with @blastdb using blastx (evalue<1e^−5^)
