# Supplementary material for: Integrating transcriptomics and metabolomics to characterise the response of Astragalus membranaceus Bge. var. mongolicus (Bge.) to progressive drought stress
Source: BMC Genomics. 2016 Mar 5;17:188. doi: 10.1186/s12864-016-2554-0 (PMC4779257; doi:10.1186/s12864-016-2554-0)
Supplement: Additional file 4: — Figure S1. A typical 600 MHz 1H NMR spectra of methanol extracts obtained from the A. mongolicus roots. (DOCX 256 kb) [file 12864_2016_2554_MOESM4_ESM.docx]

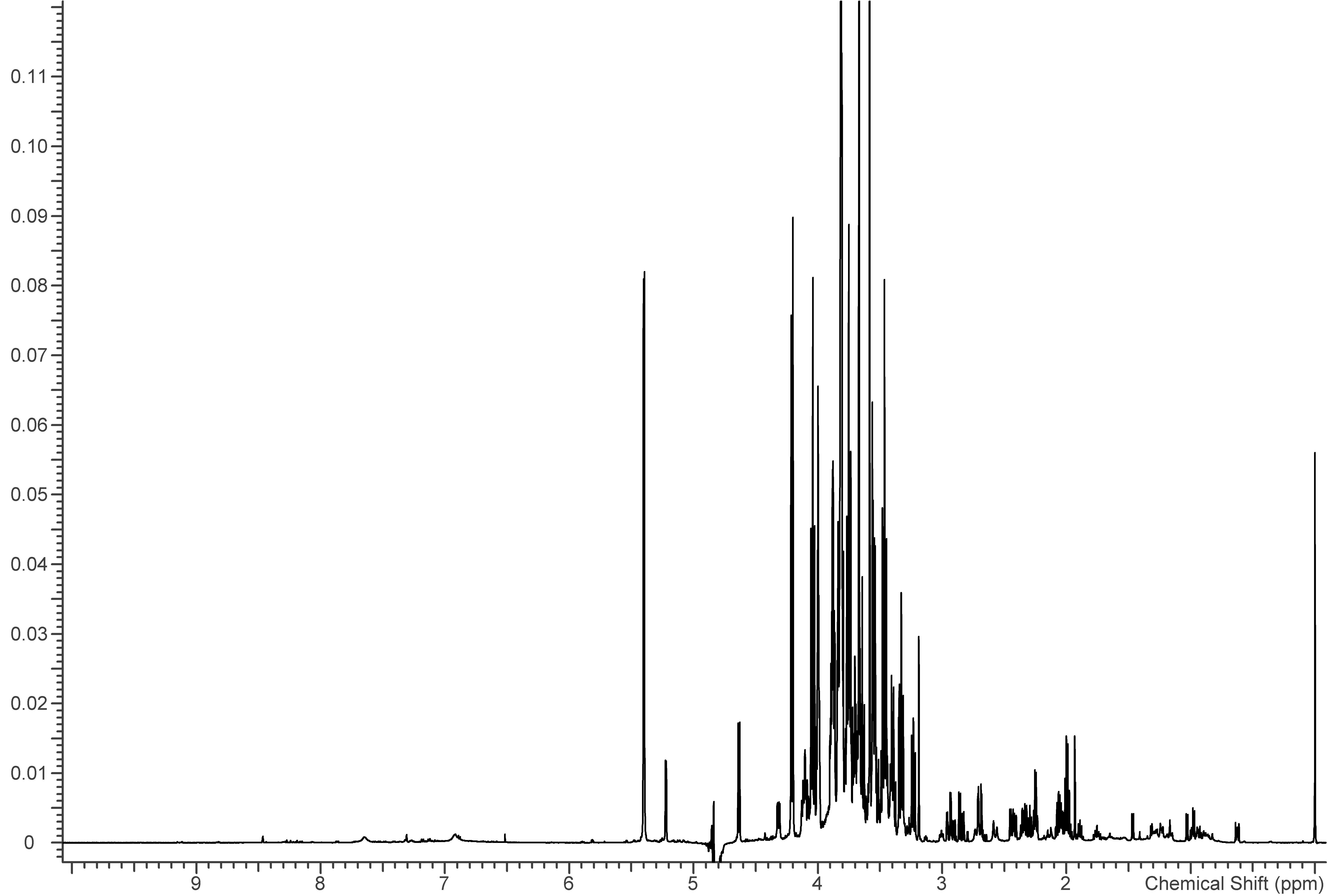


**Figure S1** Typical 600 MHz ^1^H-NMR spectra of methanol extracts from *A. mongolicus* roots. 1.4-Aminobutyrate;2.Acetate;3.Alanine;4.Arginine;5.Asparagine;6.Aspartate;7.Choline;8.Citrate;9.Ethanol;10.Ethanolamine;11.Ethyleneglycol;12.Formate;13.Fructose;14.Fumarate;15.Galactose;16.Glucose;17.Glutamate;18.Glutamine;19.Glycine;20.Hydroxyacetone;21.Isoleucine;22.Lactate;23.Leucine;24.Malate;25.Pantothenate;26.Proline;27.Pyruvate;28.Serine;29.Sucrose;30.Threonine;31.Trigonelline;32.Trimethylamine;33.Tryptophan;34.Tyrosine;35.Uridine;36.Valine;37.myo-Inositol.
