# Supplementary material for: Integrating transcriptomics and metabolomics to characterise the response of Astragalus membranaceus Bge. var. mongolicus (Bge.) to progressive drought stress
Source: BMC Genomics. 2016 Mar 5;17:188. doi: 10.1186/s12864-016-2554-0 (PMC4779257; doi:10.1186/s12864-016-2554-0)
Supplement: Additional file 6: — Table S5. The metabolite content in the A. mongolicus roots under different stages of progressive drought stress. (DOCX 21 kb) [file 12864_2016_2554_MOESM6_ESM.docx]

**Table S5** The metabolite content in the *A. mongolicus* roots under different stages of progressive drought stress

| **Metabolites** | **Metabolite quantity (mean±SE, mg/g freeze-dried plants)** | | | |
| --- | --- | --- | --- | --- |
|  | A | B | C | D |
| **Amino Acids**  **and the Derivatives** |  | | | |
| GABA | 0.65±0.08^b^ | 0.55±0.09^c^ | 1.06±0.09^a^ | 0.30±0.04^d^ |
| Alanine | 0.25±0.03^bc^ | 0.28±0.05^b^ | 0.49±0.01^a^ | 0.24±0.03^c^ |
| Arginine | 0.15±0.10 | 0.37±0.09 | 0.94±0.23 | 0.00±0.00 |
| Asparagine | 1.00±0.15^c^ | 4.39±0.68^b^ | 6.73±0.12^a^ | 1.12±0.27^c^ |
| Aspartate | 0.59±0.08^a^ | 0.46±0.09^d^ | 1.26±0.06^c^ | 0.19±0.03^b^ |
| Glutamine | 0.19±0.03 | 0.00±0.00 | 0.00±0.00 | 0.00±0.00 |
| Glycine | 0.26±0.08^c^ | 0.36±0.14^b^ | 0.55±0.12^a^ | 0.15±0.02^d^ |
| Isoleucine | 0.03±0.00^c^ | 0.14±0.03^b^ | 0.28±0.01^a^ | 0.03±0.00^c^ |
| Leucine | 0.04±0.00^c^ | 0.10±0.02^b^ | 0.13±0.02^a^ | 0.03±0.01^c^ |
| Proline | 0.21±0.03^d^ | 4.66±0.91^c^ | 8.42±0.61^b^ | 11.09±0.80^a^ |
| Serine | 0.21±0.04^b^ | 0.16±0.04^c^ | 0.28±0.04^a^ | 0.14±0.04^c^ |
| Threonine | 0.10±0.02^c^ | 0.17±0.03^b^ | 0.30±0.02^a^ | 0.06±0.01^d^ |
| Tryptophan | 0.11±0.02^b^ | 0.08±0.01^c^ | 0.15±0.02^a^ | 0.10±0.01^b^ |
| Tyrosine | 0.04±0.01^b^ | 0.13±0.02^c^ | 0.28±0.03^a^ | 0.06±0.01^b^ |
| Valine | 0.05±0.01^d^ | 0.28±0.05^b^ | 0.71±0.03^a^ | 0.09±0.01^c^ |
| Glutamate | 0.62±0.14^a^ | 0.29±0.09^b^ | 0.62±0.19^a^ | 0.56±0.20^a^ |
| **Sugars** |  | | | |
| Fructose | 5.89±0.82^c^ | 16.9±2.87^a^ | 11.8±0.34^b^ | 6.57±0.81^c^ |
| Glucose | 8.37±1.20^c^ | 22.96±4.10^b^ | 27.81±0.78^a^ | 23.13±2.02^b^ |
| Sucrose | 31.35±4.79^d^ | 64.31±12.67^c^ | 126.34±1.94^b^ | 214.92±9.39^a^ |
| Galactose | 0.22±0.05^d^ | 0.42±0.13^c^ | 0.64±0.02^b^ | 1.05±0.16^a^ |
| **Organic acids** |  | | | |
| Acetate | 0.28±0.06^c^ | 0.27±0.07^c^ | 0.35±0.07^b^ | 0.41±0.04^a^ |
| Citrate | 0.69±0.20^d^ | 0.46±0.11^c^ | 1.22±0.13^b^ | 0.95±0.22^a^ |
| Formate | 0.03±0.00^a^ | 0.01±0.00^c^ | 0.01±0.00^c^ | 0.02±0.00^b^ |
| Fumarate | 0.08±0.01^a^ | 0.10±0.02^c^ | 0.15±0.01^c^ | 0.22±0.02^b^ |
| Lactate | 0.30±0.04^a^ | 0.12±0.03^c^ | 0.03±0.00^d^ | 0.15±0.03^b^ |
| Malate | 1.01±0.20^d^ | 2.30±0.41^c^ | 4.44±0.42^a^ | 3.88±0.80^b^ |
| Pyruvate | 0.02±0.00 | 0.01±0.00 | 0.02±0.00 | 0.02±0.02 |
| **Alcohols** |  | | | |
| Ethanol | 0.02±0.00 | 0.02±0.00 | 0.02±0.00 | 0.02±0.00 |
| Ethylene glycol | 0.14±0.03^b^ | 0.10±0.02^b^ | 0.35±0.09^a^ | 0.32±0.04^a^ |
| myo-Inositol | 0.84±0.18^b^ | 0.80±0.16^b^ | 1.50±0.17^a^ | 0.86±0.10^b^ |
| **Amines** |  | | | |
| Ethanolamine | 0.05±0.01^b^ | 0.05±0.01^b^ | 0.11±0.01^a^ | 0.12±0.03^a^ |
| Trimethylamine | 0.01±0.00 | 0.01±0.00 | 0.01±0.00 | 0.01±0.00 |
| **Nucleic acid Components** |  | | | |
| Uridine | 0.17±0.01^a^ | 0.09±0.02^d^ | 0.14±0.01^b^ | 0.11±0.02^c^ |
| **Others** |  | | | |
| Choline | 0.49±0.07^c^ | 0.45±0.08^c^ | 0.90±0.02^a^ | 0.74±0.03^b^ |
| Hydroxyacetone | 0.04±0.01^b^ | 0.03±0.01^c^ | 0.05±0.01^a^ | 0.04±0.01^a^ |
| Pantothenate | 0.06±0.01^b^ | 0.06±0.01^b^ | 0.06±0.02^b^ | 0.09±0.02^a^ |
| Trigonelline | 0.01±0.00^d^ | 0.02±0.01^c^ | 0.14±0.01^b^ | 0.15±0.01^a^ |

Data is presented as mean values ± SE (n=8). The significant differences at each date of the experimental periods were presented as different letters *(P*<0.05) by one-way analysis of variance (ANOVA) using the SPSS 19.0 software. (A:control; B:water-stress for 6 days; C:water-stress for 10 days; D:water-stress for 14 days, respectively.)
